# Two Distinct Patterns of *Clostridium difficile* Diversity Across Europe Indicating Contrasting Routes of Spread

**DOI:** 10.1093/cid/ciy252

**Published:** 2018-04-06

**Authors:** David W Eyre, Kerrie A Davies, Georgina Davis, Warren N Fawley, Kate E Dingle, Nicola De Maio, Andreas Karas, Derrick W Crook, Tim E A Peto, A Sarah Walker, Mark H Wilcox, EUCLID Study Group, Kerrie A Davies, Mark H Wilcox, Georgina Davis, Christopher M Longshaw, Ed Kuijper, Lutz von Muller, Outi Lyytikainen, Silja Mentula, Fidelma Fitzpatrick, Emilio Bouza, Frederic Barbut, Monica Oleastro, Michel Delmee, Paola Mastrantonio, Torbjorn Noren, Franz Allerberger, Hanna Pituch, Maja Rupnik, Zsuzsanna Barna, Efthymia Petinaki, Otakar Nyč, Daniela Lemeni, Kate Ivanova, Elena Novakova

**Affiliations:** 1 Nuffield Department of Medicine, University of Oxford; 2 Healthcare Associated Infections Research Group, University of Leeds; 3 Public Health England, Leeds; 4 Astellas Pharma Europe, Chertsey, United Kingdom

**Keywords:** *Clostridium difficile*, transmission, healthcare, community, whole genome sequencing

## Abstract

**Background:**

Rates of *Clostridium difficile* infection vary widely across Europe, as do prevalent ribotypes. The extent of Europe-wide diversity within each ribotype, however, is unknown.

**Methods:**

Inpatient diarrheal fecal samples submitted on a single day in summer and winter (2012–2013) to laboratories in 482 European hospitals were cultured for *C. difficile*, and isolates the 10 most prevalent ribotypes were whole-genome sequenced. Within each ribotype, country-based sequence clustering was assessed using the ratio of the median number of single-nucleotide polymorphisms between isolates within versus across different countries, using permutation tests. Time-scaled Bayesian phylogenies were used to reconstruct the historical location of each lineage.

**Results:**

Sequenced isolates (n = 624) were from 19 countries. Five ribotypes had within-country clustering: ribotype 356, only in Italy; ribotype 018, predominantly in Italy; ribotype 176, with distinct Czech and German clades; ribotype 001/072, including distinct German, Slovakian, and Spanish clades; and ribotype 027, with multiple predominantly country-specific clades including in Hungary, Italy, Germany, Romania, and Poland. By contrast, we found no within-country clustering for ribotypes 078, 015, 002, 014, and 020, consistent with a Europe-wide distribution. Fluoroquinolone resistance was significantly more common in within-country clustered ribotypes (*P* = .009). Fluoroquinolone-resistant isolates were also more tightly clustered geographically with a median (interquartile range) of 43 (0–213) miles between each isolate and the most closely genetically related isolate, versus 421 (204–680) miles in nonresistant pairs (*P* < .001).

**Conclusions:**

Two distinct patterns of *C. difficile* ribotype spread were observed, consistent with either predominantly healthcare-associated acquisition or Europe-wide dissemination via other routes/sources, for example, the food chain.

## Teaser

Whole-genome sequencing of *Clostridium difficile* across Europe demonstrates 2 distinct transmission patterns. Healthcare-associated ribotypes showed genetic clustering by country and region, and widespread fluoroquinolone resistance. However, multiple common ribotypes showed no within-country clustering, consistent with Europe-wide dissemination via nonhealthcare routes/sources.

*Clostridium difficile* can spread readily in healthcare facilities [1] and has caused large-scale healthcare-associated epidemics in the 1990s [2] and 2000s [3], particularly with the ribotype 027/NAP1 strain [4]. However, with enhanced hospital infection prevention and control [5], <40% of cases are estimated to be acquired from other symptomatic cases, using whole-genome sequencing in Oxford (35%) [6], Leeds (35%) [7], and Liverpool (37% of ribotype 027 cases) [8], United Kingdom, and 6 hospitals in England (≤31%) [9], and using multilocus variable number tandem repeat analysis in Pennsylvania (30%) [10]. Antimicrobial restriction has also probably played a key role in reducing *C. difficile* infection (CDI) [11]. For example, CDIs with clindamycin-resistant [12, 13] and fluoroquinolone-resistant [14] strains declined in the United States and United Kingdom, respectively, after altered antibiotic prescribing.

*C. difficile* is probably also acquired from other sources, including patients colonized with toxigenic *C. difficile* with diarrhea of another cause [15] or asymptomatic patients [10, 16–18], asymptomatically colonized children [19, 20], domestic and production animals, and the food chain [21]. Strains of *C. difficile* may have particular ecological niches and are preferentially transmitted in different environments. For example, ribotype 027/NAP1 has successfully spread in healthcare settings worldwide [4]. Ribotype 001/NAP2 is also found predominantly in hospitals, for example, in the United Kingdom [22] and Canada [23]. Ribotype 018 was highly transmissible in an Italian hospital study [24], and the closely related ribotype 356 was the most common strain in a survey of Italian hospitals [25]. In the United States, ribotype 027/NAP1 is found in healthcare- and community-associated CDI but still has the greatest ratio of healthcare-associated to community-associated cases among the commonly circulating strains [26].

Other strains have nonhealthcare reservoirs; for example, ribotype 078 has been found in pigs and cattle [21, 27] with close genetic relatedness on whole-genome sequencing between isolates from pigs, healthy farmers, and CDI case patients in the Netherlands [27]. Ribotype 078 infections are more likely to be community acquired and affect younger patients [28]. In the United States in 2011, ribotype 078/NAP7, ribotype 002/NAP6, and to a lesser extent ribotype 020/014/NAP4, occurred predominantly in community-associated cases, based on surveillance definitions [26]. Community-associated ribotype strains may be widely distributed in the environment; for example, ribotypes 014, 015, and 078 were all identified at Swiss wastewater treatment plants [29].

A point-prevalence survey (EUCLID) has demonstrated marked Europe-wide variation in both CDI and testing rates [30] and prevalent ribotypes [31]. We determined the extent of Europe-wide diversity within the 10 most prevalent ribotypes from that study to investigate whether different modes of transmission could plausibly occur across these ribotypes. We also investigated the relationship between geographic distribution and antimicrobial susceptibilities to explore potential drivers of healthcare- and community-associated infections.

## METHODS

### Study Design and Samples

Samples were obtained during a European, multicentre, prospective, point-prevalence study of hospital inpatients with diarrhea (EUCLID) [30]. The study included 20 European countries, each with a national coordinating laboratory. National coordinators selected the 482 participating hospitals to cover all major geographic regions, at a rate of 1 per 1 million population. Ethical approval was granted in the Netherlands, Sweden, and Slovenia; the remaining countries did not require such approval because the study was classed as surveillance.

All inpatient diarrheal samples submitted to the microbiology laboratory of the participating hospitals on 2 sampling days (1 in winter [December 2012 through January 2013], and 1 in summer [July–August 2013]) were eligible, irrespective of the original test(s) requested. Samples were included from all patients >2 years old currently occupying a hospital bed, including patients in the emergency department but excluding outpatient clinic attendees. Only 1 sample per patient on each sampling day was included. All samples were tested for CDI at the national coordinating laboratory using the same standardized method (*C. DIFF QUIK CHEK COMPLETE*; Techlab); those that were glutamate dehydrogenase or toxin A/B enzyme immunoassay positive were also cultured and isolates sent to the Leeds General Infirmary microbiology laboratory for polymerase chain reaction ribotyping [32]. A total of 7297 inpatient diarrheal samples were tested at 482 European hospitals [30], resulting in 1211 isolates sent for ribotyping, with distribution and characteristics (eg, age) as described elsewhere [31].

### Sequencing

Isolates from the 10 most prevalent toxigenic ribotypes underwent whole-genome sequencing. DNA was extracted from subculture of a single colony and sequenced using the Illumina HiSeq 2500 system. Sequence data were processed as described elsewhere [6, 33], mapping reads to the *C. difficile* 630 reference genome (AM180355.1), except for isolates from ribotypes 027 and 078, whose reads were mapped to CD196 (NC_013315.1) and M120 (FN665653.1), respectively. Sequences were compared using single-nucleotide polymorphisms (SNPs), obtaining differences between sequences from maximum likelihood phylogenies corrected for recombination [34]. BEAST (version 2.4.3) [35] and BASTA (version 2.3.0) [36] software were used to reconstruct time-scaled phylogenies and to infer the geographic location of each ancestral lineage.

A strength of this approach is that it is possible to reconstruct past geographic locations and patterns of geographic spread for each lineage, despite sequencing isolates at only 2 time points. Antimicrobial resistance determinants for fluoroquinolones, tetracycline, clindamycin/macrolides, aminoglycosides, and fidaxomicin were identified from mapped data and de novo assemblies. Further details are provided in the Supplementary Methods and Supplementary Table 1. Sequencing quality metrics are provided in Supplementary Table 2. Sequence data generated are available from the NCBI Sequence Read Archive under BioProject PRJNA398458 (see Supplementary Table 3 for study sample metadata).

### Country-based Clustering

For each ribotype, the median number of SNPs between isolates within a country (SNP_within_), between sequences across different countries (SNP_across_), and overall were calculated. Country-based sequence clustering was assessed using the SNP_within_/SNP_across_ ratio; values closer to 0 indicate greater country-based clustering. To determine whether observed SNP_within_/SNP_across_ ratios were compatible with the null hypothesis of no country-based clustering, for each ribotype, the country labels of each tip of the phylogenetic tree were permuted 1000 times. For each permutation, the ratio was calculated. Country-based clustering was supported if the observed ratio was less than the 2.5% quantile of the distribution of permuted ratios. Because both the overall diversity within each ribotype and the number of samples varied, SNP_within_/SNP_across_ ratios are not directly comparable between ribotypes. A similar approach was used to determine, by ribotype, whether there was evidence of within-hospital clustering in sequences obtained from the same country.

## RESULTS

**Figure 1.**
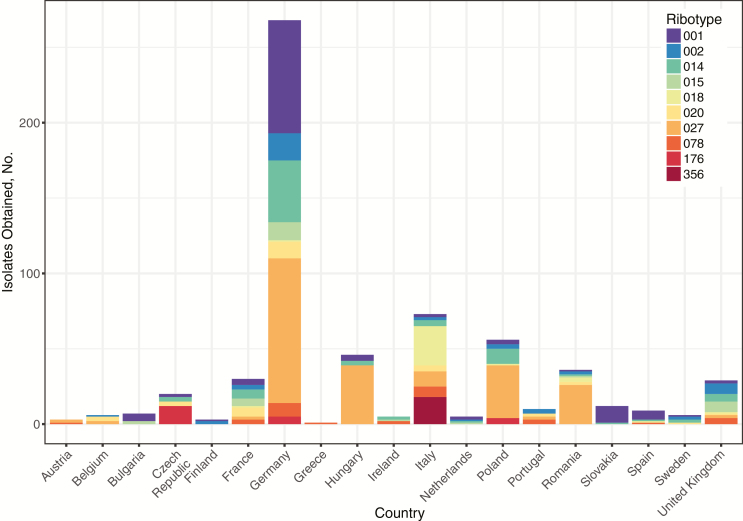
Distribution of isolates from sequenced ribotypes by country. Study hospitals were recruited at a rate of 1 per million head of population; hence, differences in total isolates obtained between countries represent differences in within-country testing rates and incidence of *Clostridium difficile* infection (see Table 2).

Of 1211 isolates obtained in 2012–2013 from 482 European hospitals, 678 were from the 10 most prevalent ribotypes (Figure 1). Of these, 624 (92%) were retrieved and successfully whole-genome sequenced from ribotypes 027 (n = 216), 001/072 (n = 119), 014 (n = 80), 002 (n = 44), 018 (n = 34), 020 (n = 33), 078 (n = 31), 015 (n = 29), 176 (n = 21), and 356 (n = 17) (Table 1). Sequenced isolates came from 19 countries: Germany (n = 268), Italy (n = 72), Poland (n = 56), Hungary (n = 46), Romania (n = 36), France (n = 30), United Kingdom (n = 29), Czech Republic (n = 20), Slovakia (n = 12), Portugal, Spain, Bulgaria, Belgium, Sweden, Ireland, the Netherlands, Austria, Finland, and Greece (each n = 1–10; see Table 2 for per-country CDI testing-rate/incidence).

**Table 1.** Extent of Within-country Clustering by Ribotype

Ribotype	Isolates, No.	Isolates Sequenced, No. (%)	Countries Where Ribotype Was Found, No.	SNPs Between Isolates, Overall Median (IQR)	TMRCA (95% HPD)	SNP_across_ Median (IQR)	SNP_within_ Median (IQR)	SNP_within_/SNP_across_ Ratio	Expected Ratio, 95% CI^b^	Compatible With Random European Distribution	Distance Between Hospitals for Most Closely Related Isolates, Median (IQR), Miles^a^
020	37	33 (89)	9	43 (34–64)	1955 (1920–1986)	52 (34–104)	42 (34–60)	1.25	.84–1.24	Yes	542 (256–796)
014	86	80 (93)	13	146 (66–172)	1963 (1924–1995)	147 (59–173)	146 (68–171)	1.01	.87–1.09	Yes	347 (198–608)
002	48	44 (92)	11	73 (61–97)	1955 (1922–1986)	69 (59–88)	75 (62–100)	0.92	.88–1.05	Yes	443 (191–817)
015	30	29 (97)	7	298 (54–366)	1857 (1757–1943)	296 (29–364)	346 (189–433)	0.86	.84–1.18	Yes	280 (176–574)
078	37	31 (84)	9	54 (34–148)	1721 (1556–1872)	56 (29–129)	54 (35–152)	1.04	.74–1.43	Yes	424 (266–812)
027	223	216 (97)	10	71 (50–110)	1949 (1913–1977)	49 (17–71)	82 (60–125)	0.60	.93–1.07	No	21 (0–189)
001/072	134	119 (89)	14	201 (41–273)	1851 (1754–1934)	37 (23–199)	253 (173–322)	0.15	.70–1.31	No	184 (0–351)
176	25	21 (84)	3	15 (7–19)	1993 (1977–2004)	6 (4–8)	19 (16–20)	0.31	.65–1.23	No	36 (0–198)
018	36	34 (94)	7	39 (22–88)	1787 (1638–1910)	28 (17–38)	92 (83–285)	0.30	.40–1.56	No	189 (53–636)
356	22	17 (77)	1	9 (5–12)	2008 (2004–2011)	9 (5–12)	…	…	…	No	133 (57–248)

Abbreviations: CI, confidence interval; HPD, highest posterior density interval; IQR, interquartile range, SNP, single-nucleotide polymorphism; SNP_across_, SNPs between isolates across different countries; SNP_within_, SNPs between isolates within a country; TMRCA, time of most recent common ancestor.

^a^To calculate the number of miles between the most closely related isolates, within each ribotype, a minimum spanning tree was constructed using SNP distances as edge weights and isolates as nodes. The properties of the edges (SNPs and miles) of the minimum-spanning tree were analyzed, such that each isolate but 1 contributes 1 data point (see Figure 5).

^b^CIs were based on permutation tests (see Methods).

**Table 2.** Mean Measured Clostridium difficile Infection Rate and Testing Rate per 10 000 Bed Days^a^

Country	Mean Measured Rate per 10 000 Bed d	
	CDI Rate	Testing Rate
Austria	7.2	102.0
Belgium	5.4	95.4
Bulgaria	32.2	1.0
Czech Republic	18.9	140.0
Finland	12.6	102.2
France	3.8	38.8
Germany	24.8	137.2
Greece	3.5	56.4
Hungary	17.7	71.7
Ireland	6.1	274.5
Italy	11.9	67.7
The Netherlands	6.1	88.1
Poland	38.9	135.0
Portugal	17.0	72.3
Romania	93.4	35.4
Slovakia	16.9	84.2
Spain	10.4	95.2
Sweden	11.9	71.8
United Kingdom	5.7	115.8
Europe	18.1	93.9

Abbreviation: CDI, *Clostridium difficile* infection.

^a^Mean values are averages for winter and summer sampling days.

### Ribotype Diversity Within and Between Countries

Within ribotypes, the overall median number of SNPs between isolates ranged from 9 to 298 (Table 1). Using time-scaled phylogenies, the most recent common ancestor for most ribotypes was estimated to date from the 18th to the mid-20th century, with the exception of ribotypes 176 and 356, which emerged more recently, in 1993 (95% highest posterior density interval [HPD], 1977–2004) and 2008 (2004–2011), respectively. The latter recently evolved from ribotype 018 in Italy (Supplementary Figure 1).

**Figure 2.**
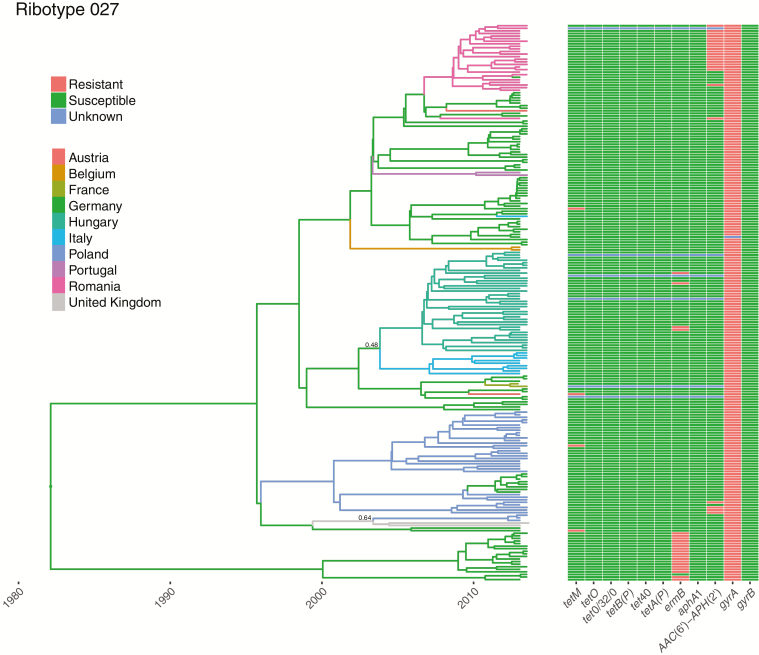
Ribotype 027 phylogeny with ancestral country reconstruction and antimicrobial resistance determinants. A time-scaled phylogeny, dated in years, is shown with the branches colored by the inferred country that the ancestral strains were present in over time. The thickness of the lines indicates the level of posterior support for the assigned countries; where the posterior support is <0.8, a numeric value is provided. Note that the choice of inferred ancestor is limited to those sampled. The right-hand panel shows for each strain the presence of determinants of tetracycline (*tetM*, *tetO*, *tetB[P]*, *tet0/32/0*, *tet40*, *tetA[P]*), clindamycin and macrolide (*ermB*), aminoglycoside (*aphA1*, *AAC[6’]–APH[2’]*) and fluoroquinolone (*gyrA* and *gyrB* mutations) resistance. Where de novo assemblies or base calls were not of sufficient quality to make reliable assignations, the result is denoted as unknown.

Five ribotypes had evidence of clear within-country clustering: ribotype 356, found only in Italy; ribotype 018, predominantly isolated in Italy; ribotype 176, with distinct Czech and German clades; ribotype 001/072 including distinct German, Slovakian, and Spanish clades; and ribotype 027, with multiple predominantly country-specific clades, including in Hungary, Italy, Germany, Romania, and Poland (Figure 2 and Supplementary Figures 1–3). For these ribotypes, the SNP_within_/SNP_across_ ratio was significantly lower than expected by chance (Table 1); for example, for ribotype 027 the ratio was 0.60, versus an expected value of .93–1.07 without any country-based clustering (95% confidence interval from permutation test).

**Figure 3.**
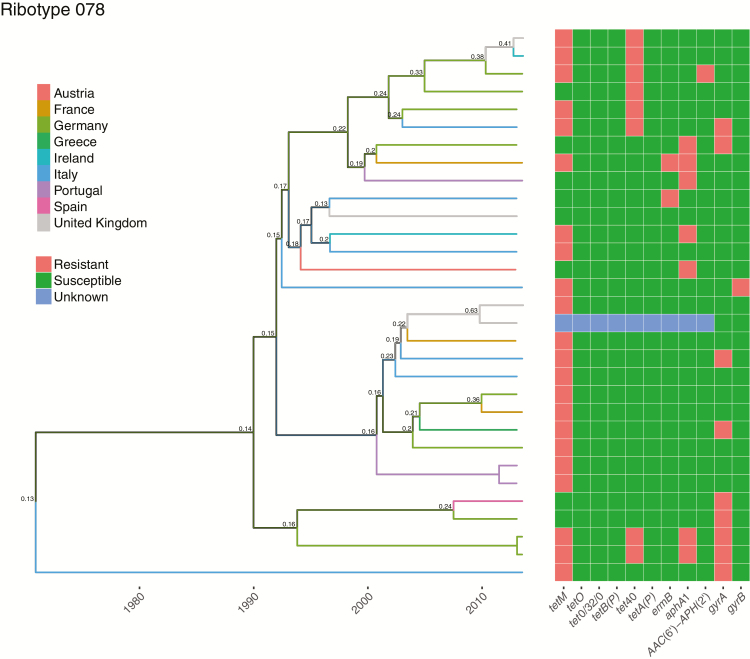
Ribotype 078 phylogeny with ancestral country reconstruction and antimicrobial resistance determinants. A time-scaled phylogeny, dated in years, is shown with the branches colored by the inferred country that the ancestral strains were present in over time. The thickness of the lines indicates the level of posterior support for the assigned countries; where the posterior support is <0.8, a numeric value is provided.

By contrast, there was no evidence of within-country clustering for ribotypes 078, 015, 002, 014, and 020 (Figure 3 and Supplementary Figures 4–7), consistent with recent Europe-wide distribution. For these ribotypes, the SNP_within_/SNP_across_ ratio was approximately 1 and fell within the 95% confidence interval expected by chance, assuming no country-based clustering (Table 1).

For most ribotypes the median number of SNPs between sequences from the same hospital was less than that between sequences within countries—for example, 8 (interquartile range [IQR], 3–26) versus 49 (17–71) SNPs for ribotype 027 (Table 3), compatible with onward transmission within institutions. For ribotypes showing within-country clustering, based on permutation tests, there was evidence of smaller-scale within-institution clustering for ribotypes 027, 001/072, and 018. There was limited local and national diversity within ribotypes 176 and 356. Among ribotypes compatible with a recent pan-European distribution, ribotypes 014, 002, and 015 had lower median numbers of within-hospital than within-country SNPs, but ribotypes 020 and 078 did not. The median numbers of SNPs within countries between pairs of sequences not from the same hospital were very similar to those within countries overall, demonstrating that, where observed, country-based clustering is not just driven by hospital-based clustering.

**Table 3.** Extent of Within-hospital Clustering by Ribotype

Ribotype	SNPs Between Isolates, Median (IQR)			Within-hospital/Different Hospital Median SNP Ratio	Expected Ratio, 95% CI^a^	Compatible With Hospital-based Clustering
	Within Country	Within Hospital	Within Country, Different Hospital			
020	52 (34–104)	40 (31–105)	54 (35–104)	0.74	.51–2.04	No
014	147 (59–173)	32 (0–137)	148 (60–173)	0.21	.42–1.17	Yes
002	69 (59–88)	3 (0–21)	69 (60–88)	0.05	.65–1.36	Yes
015	296 (29–364)	2 (0–2)	296 (41–365)	0.01	.14–1.47	Yes
078	56 (29–129)	125 (1–250)	56 (32–128)	2.26	.15–3.86	No
027	49 (17–71)	8 (3–26)	50 (19–71)	0.16	.42–0.56	Yes
001/072	37 (23–199)	9 (4–31)	38 (24–201)	0.23	.70–1.09	Yes
176	6 (4–8)	7 (2–8)	6 (4–8)	1.17	.66–1.17	Limited total diversity
018	28 (17–38)	7 (4–11)	29 (17–38)	0.23	.52–1.38	Yes
356	9 (5–12)	7 (0–7)	9 (5–12)	0.78	.22–1.56	Limited total diversity

Abbreviations: CI, confidence interval; IQR, interquartile range; SNP, single-nucleotide polymorphism.

^a^CIs were based on permutation tests (see Methods).

### Country-based Clustering and Antimicrobial Resistance

**Figure 4.**
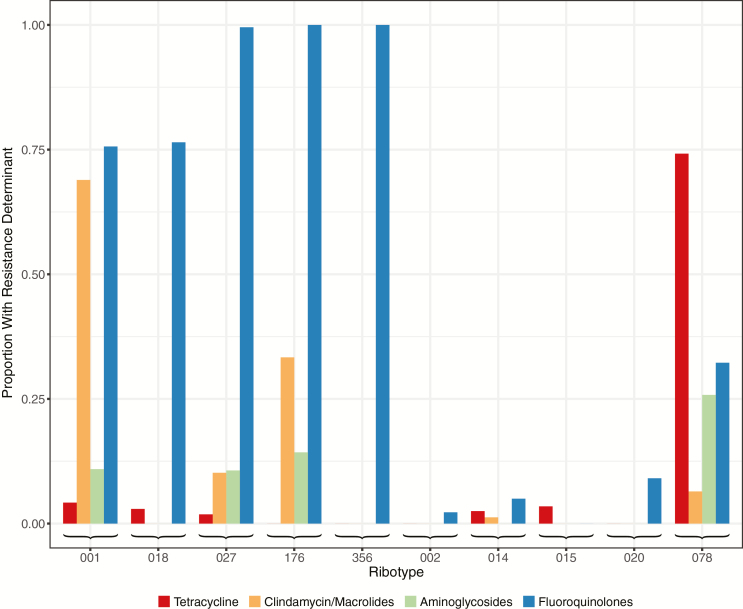
Relationship between ribotypes and presence of resistance determinants.

There was a strong association between within-country clustering and the presence of fluoroquinolone resistance determinants (Figure 4). The proportion of isolates with fluoroquinolone resistance was significantly higher in the 5 ribotypes showing within-country clustering than in the remaining 5 ribotypes (rank sum *P* = .009). There was no association between clustering and resistance determinants for 3 antimicrobials, tetracyclines (*P* = .75), aminogylcosides (*P* = .41), and clindamycin/macrolides (*P* = .22). No isolates had previously described fidaxomicin resistance determinants.

All assessable isolates from ribotypes 027, 176, and 356 had fluoroquinolone resistance mutations. Only a subset of ribotype 018 and 001/072 isolates had resistance mutations allowing the date of the most recent common ancestor of each resistant clade to be estimated. In ribotype 018, fluoroquinolone resistance was estimated to have emerged in 2001 (95% HPD, 1993–2008), followed by a clonal expansion restricted to Italy, from which ribotype 356 descended (Supplementary Figure 1). In ribotype 001/072 fluoroquinolone resistance was strongly associated with the presence of *ermB,* conferring resistance to clindamycin and macrolides. Country-specific clonal expansions of fluoroquinolone/clindamycin/macrolide-resistant ribotype 001/072 strains occurred in Germany from 2000 (95% HPD, 1992–2007), in Slovakia from 2006 (2001–2010), and in Spain from 1994 (1981–2006) (Supplementary Figure 2).

### Geographic Distance and Genetic Clustering

**Figure 5.**
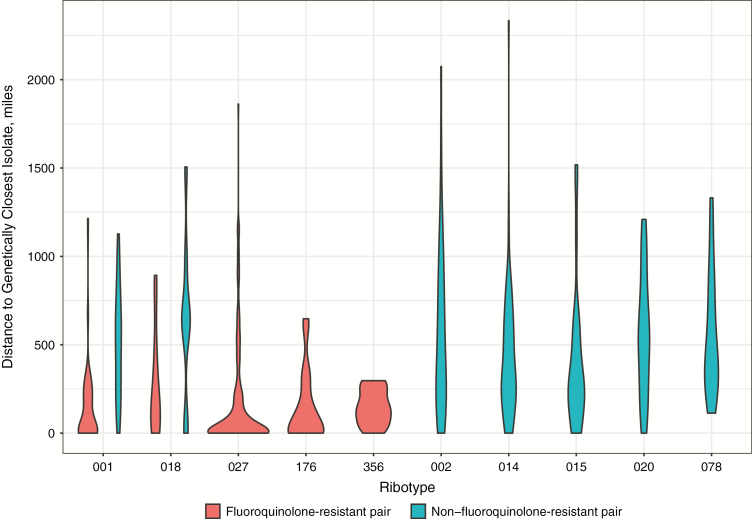
Geographic distance to genetically closest isolate, by ribotype and fluoroquinolone resistance. Within each ribotype, a minimum spanning tree was constructed using genetic distances measured in single-nucleotide polymorphisms (SNPs) as edge weights and isolates as nodes. The properties of the edges of the minimum-spanning tree are plotted, such that each isolate but 1 contributes 1 data point. Fluoroquinolone-resistant pairs of isolates were both fluoroquinolone resistant, and non–fluoroquinolone-resistant pairs were either both or only 1 susceptible. One pair of ribotype 014 isolates were both fluoroquinolone resistant, as were 2 pairs of ribotype 078 isolates; however, these are insufficient to estimate a kernel density to plot.

Ribotypes that clustered by country also showed evidence of tighter geographic clustering by region (Figure 5 and Table 1). The median geographic distance between each isolate and the next most closely genetically related isolate (using minimum spanning trees to avoid double counting; see legend to Figure 5) was 43 (IQR, 0–213) miles in fluoroquinolone-resistant pairs versus 421 (204–680) miles in nonresistant pairs (rank sum *P* < .001). By ribotype, the geographic distance between the most closely genetically related isolates was lower for fluoroquinolone-resistant pairs: 21 (IQR, 0–189), 36 (0–198), and 133 (57–248) miles in fluoroquinolone-resistant ribotypes 027, 176 and 356 respectively. In ribotype 001/072, the median distance was 56 (IQR, 0–221) versus 512 (230–706) miles in fluoroquinolone-resistant versus nonresistant pairs, respectively (*P* < .001), and in ribotype 018, it was 160 (53–445) versus 642 (461–750) miles (*P* = .08). By contrast, median distances to the most genetically related isolates in ribotypes without country-based clustering were larger, at 443 (IQR, 191–817), 347 (198–608), 280 (176–564), 542 (256–796), and 424 (266–812) miles in ribotypes 002, 014, 015, 020, and 078, respectively.

## DISCUSSION

Here we show 2 distinct patterns of *C. difficile* ribotype spread. Ribotypes previously associated with healthcare-based transmission, including ribotypes 027 and 001/072, demonstrated evidence of country, regional, and within-hospital clustering. This geographic structure was preserved over several years of *C. difficile* evolution in each ribotype. In many instances the most plausible source of all cases of a particular ribotype, or subclade of a ribotype, in a country could be explained by a single imported case, suggesting that intercountry transmission of *C. difficile* with these ribotypes is unusual.

By contrast, other ribotypes (002, 014, 015, 020, and 078) showed no evidence of country-based clustering, and in many cases the most closely genetically related strains were separated by several hundred miles. This suggests that these ribotypes are widely disseminated across Europe, and sustained local transmission is less common than with the healthcare-associated ribotypes described above. It is likely that there is still some secondary transmission of these ribotypes within hospitals. Although by sampling on only 2 days 6 months apart this study did not fully capture the extent of local transmission, we did find evidence of within-hospital clustering in ribotypes 014, 002, and 015.

However, the overall pattern observed is consistent with a dominant route of spread other than healthcare. The association of ribotypes 078 [27] and 014 [37] with pig farming, and previous findings of *C. difficile* in food, raises the possibility that the food chain may be an important vector for these ribotypes. The most recent ancestor of pairs of closely related samples from these ribotypes was often 5–10 years earlier. Therefore, the spread of these strains across Europe may have occurred several years previously with persistence in local reservoirs; alternatively, more recent spread may have occurred from a reservoir supporting a more diverse *C. difficile* population than that found in a single human host.

We also found that country-based clustering of *C. difficile*, and therefore probably healthcare-associated transmission, was strongly associated with fluoroquinolone resistance, not just in ribotype 027 but also in ribotypes 001/072, 018/356, and 176. In ribotype 001/072, fluoroquinolone resistance was found together with *ermB* conferring resistance to clindamycin and macrolides, possibly reflecting fluoroquinolone resistance acquisition by an already successfully healthcare-adapted clindamycin-resistant clone or vice versa; otherwise, there was no evidence of associations between country-based clustering and resistance to other antimicrobials, including tetracycline and aminoglycosides. We found no evidence of fidaxomicin resistance. *Clostridium difficile* is widely resistant to cephalosporins; therefore, although cephalosporins can be a risk factor for some infections, their use is unlikely to differentially affect the transmission of some ribotypes.

Supporting the importance of fluoroquinolone use as a driver of healthcare-associated CDI, Dingle et al [14] demonstrated that declining CDI incidence in England this millennium can be accounted for by falls in fluoroquinolone-resistant *C. difficile*, while levels of fluoroquinolone-susceptible *C. difficile* have remained largely unchanged. Clonal expansions, representing rapid successful transmission, followed the acquisition of fluoroquinolone resistance in 2 ribotype 027 lineages [4]. Here we provide additional examples of clonal expansions following acquisition of fluoroquinolone resistance in ribotypes 018 and 001/072 dating from the 1990s onward, consistent with the launch of fluoroquinolones in Europe in 1987.

In contrast to previous findings of He et al [4], who reported frequent and extensive long-range geographic transmission within the United Kingdom of ribotype 027, transmission events between European countries were relatively rare. They typically involved geographically adjacent countries, although we observed probable spread from the United Kingdom to Poland as well, possibly reflecting the population flows between these countries. We were able to observe marked European country-based clustering of some ribotypes because a sufficiently large number of isolates was obtained from each country in our study.

A strength of our study is its unbiased sampling of *C. difficile* across Europe, with samples obtained on a single day in summer and winter, and the number of hospitals from each country based on its population. However, because only 2 single time points were captured, analyses will tend to underestimate the extent of local secondary transmission for all ribotypes, because this results in genetically related cases that typically occur close in time but are not often sampled on the same day.

Other study limitations include the dependence on the rate of submission of diarrheal samples to hospital laboratories. Despite efforts to account for some underreporting by testing all samples, irrespective of the original test requested, patient testing rates varied widely between countries (Table 2). This may have affected the relative prevalence of different ribotypes, but our conclusions regarding within-country clustering should be robust to this. In addition, the reconstruction of the ancestral country for each lineage is only an approximation, given that, owing to limited sample size, we had to assume the same effective bacterial population size in each country, and the same rates of exchange of strains between all countries. The study is also limited by only considering transmission between countries as discrete entities. The study was insufficiently powered to explore regional transmission, including the impact of referral networks, hospital characteristics, and differing patient catchments. These could be investigated in larger national studies, as has been done for other healthcare-associated pathogens, for example, methicillin-resistant *Staphylococcus aureus* [38].

Our findings have important implications for *C. difficile* surveillance and control. Its transmission may be more heterogeneous than is usually assumed, with different interventions required for predominantly healthcare-associated and community-associated lineages. Surveillance programs may need to consider routine use of typing to allow the incidence of commonly healthcare-associated strains to be followed independently of community-associated strains. Although this is theoretically possible with surveillance definitions based on the timing of the last healthcare exposure, recent healthcare exposure may merely be a marker of exposure to a precipitant of symptoms (eg, antibiotics), rather than the location of *C. difficile* acquisition.

Measures to control healthcare-associated *C. difficile* are well established. However, it is less clear how to control the spread of ribotypes that are widely distributed across Europe. Further studies are required to better understand potential vectors for transmission, for example, contaminated food or use of animal-based fertilizers. In principal, better antimicrobial stewardship should reduce the risk of all types of CDI, by reducing patients’ exposure to provocative antibiotics. However, because *C. difficile* antimicrobial resistance is associated with country-based clustering, stewardship may have a greater effect on healthcare-associated strains. If the nonclustered ribotypes are found very widely in the environment, prevention of exposure may be particularly challenging, and other strategies in addition to antimicrobial stewardship may need to be explored to reduce infections, including vaccination or prophylaxis during at-risk periods, for example, around healthcare exposures.

## Supplementary Data

Supplementary materials are available at *Clinical Infectious Diseases* online. Consisting of data provided by the authors to benefit the reader, the posted materials are not copyedited and are the sole responsibility of the authors, so questions or comments should be addressed to the corresponding author.

Supplementary Material

## Notes

***EUCLID study group.*** European coordinators: Kerrie A. Davies (Leeds Teaching Hospitals NHS Trust, United Kingdom), Mark H. Wilcox (University of Leeds), and Georgina Davis (University of Leeds ). European coordinator and representative of sponsor: Christopher M. Longshaw (Astellas Pharma Europe, Chertsey, UK). National coordinators: Ed Kuijper (Leiden University Medical Centre, the Netherlands), Lutz von Muller (Saarland University Medical Centre, Germany), Outi Lyytikainen and Silja Mentula (National Institute of Health and Welfare, Helsinki, Finland), Fidelma Fitzpatrick (Health Protection Surveillance Centre and Beaumont Hospital, Dublin, Ireland), Emilio Bouza (Hospital General Universitario ‘Gregorio Maranon’, Madrid, Spain), Frederic Barbut (Saint-Antoine Hospital, France), Monica Oleastro (National Institute of Health, Lisbon, Portugal), Michel Delmee (University of Louvain, Belgium), Paola Mastrantonio (Istituto Superiore di Sanita, Rome, Italy), Torbjorn Noren (Orebro University Hospital, Sweden), Franz Allerberger (Austrian Agency for Health and Food Safety, Vienna, Austria), Hanna Pituch (Department of Medical Microbiology, Medical University of Warsaw, Poland), Maja Rupnik (National Laboratory for Health, Environment and Food, Maribor, Slovenia), Zsuzsanna Barna (National Centre for Epidemiology, Budapest, Hungary), Efthymia Petinaki (University Hospital of Larissa, Greece), Otakar Nyč (University Hospital FN Motol, Prague, Czech Republic), Daniela Lemeni (Cantacuzino Institute, Bucharest, Romania), Kate Ivanova (National Centre of Infectious and Parasitic Diseases, Sofia, Bulgaria), and Elena Novakova (Jessenius Faculty of Medicine in Martin Comenius University in Bratislava, Slovakia).

***Financial support.*** This work was supported by Astellas Pharma. The EUCLID study was initiated and wholly supported financially by Astellas Pharma and endorsed by the European Society of Clinical Microbiology and Infectious Diseases Study Group for *Clostridium difficile*. D. W. E. is a clinical lecturer for the National Institute for Health Research.

***Potential conflicts of interest.*** A. K. is a full-time employee of Astellas Pharma. M. H. W. has received consulting fees from Actelion, Astellas, bioMerieux, Cambimune, Da Volterra, MedImmune, Merck, Meridian, Pfizer, Qiagen, Sanofi-Pasteur, Seres, Summit, Synthetic Biologics, and Valneva, lecture fees from Alere, Astellas, Merck, and Pfizer, and grant support from Actelion, Astellas, bioMerieux, Da Volterra, Merck, Sanofi-Pasteur, Seres, and Summit. All other authors report no potential conflicts.
