# Supplementary material for: Two Distinct Patterns of Clostridium difficile Diversity Across Europe Indicating Contrasting Routes of Spread
Source: Clin Infect Dis. 2018 Apr 6;67(7):1035–44. doi: 10.1093/cid/ciy252 (PMC6137122; doi:10.1093/cid/ciy252)
Supplement: Supplementary Material [file ciy252_suppl_supplementary_material.docx]

# Two distinct patterns of *Clostridium difficile* diversity across Europe indicates contrasting routes of spread: Supplementary Material

## Supplementary methods

Sequencing

Isolates from the 10 most prevalent toxigenic ribotypes underwent whole-genome sequencing. DNA was extracted from subculture of a single colony and sequenced using Illumina HiSeq2500, generating 100 base-pair paired-end reads. Sequence data were processed as previously,^1,2^ mapping reads to the *C. difficile* 630 reference genome (AM180355.1), except ribotype-027 and ribotype-078 isolates whose reads were mapped to CD196 (NC_013315.1) and M120 (FN665653.1) respectively.

Sequences were compared using single nucleotide polymorphisms, SNPs, obtaining differences between sequences from maximum likelihood phylogenies constructed from mapped read data using PhyML version 3.1^3^ (with generalized time-reversible substitution model and “BEST” tree topology search algorithm), corrected for recombination using ClonalFrameML version 1.25^4^ (with default settings). Sequence reads were also assembled *de novo* with Velvet*,*^5^ using VelvetOptimiser. Antimicrobial resistance determinants for fluoroquinolones, tetracycline, clindamycin/macrolides, aminoglycosides, and fidaxomicin were identified from mapped data (*gyrA*, *gyrB*, *rpoB*, *rpoC*, CD22120 point mutations/truncation) and using BLAST searches of *de novo* assemblies (*tet*, *ermB*, *aphA1*, and *AAC(6’)-APH(2’)* genes [details in table S1]). Allowing gene matches split across two or more contigs, genes were considered present where nucleotide identity was >80% along the entire gene length. Excepting *AAC(6’)-APH(2’)*, all genes identified had ≥97% nucleotide identity, and only 6/8514 blast searches contained matches with <80% identity. De novo assemblies had to contain 3x10^6^ to 4.5x10^6^ bases within contigs ≥1k in length, otherwise the resistance genotype was defined as unknown. Table S2 provides sequencing quality metrics for reference-based mapping and *de novo* assemblies, by ribo­type.

Phylogeopraphic analysis

BEAST version 2.4.3^6^ and BASTA version 2.3.0^7^ were used to reconstruct time-scaled phylogenies, and to infer the geographic location of each ancestral lineage. Input mapped sequence alignments had recombination removed using ClonalFrameML outputs. Three or more independent runs, each with ≥10 million iterations, were combined to determine the final trees, after discarding the first ≥1 million iterations of each run as burn-in, and ensuring similar convergence. A HKY substitution model was used. The mutation rate prior point estimate and uncertainty was set as previously estimated (log-normal prior, mean=-15.6, standard deviation=0.3).^1^ Given the relatively small number of samples from each country, a single parameter was estimated for the within-country effective bacterial population size for all countries (log-normal prior, mean=4, standard deviation=8), and similarly a single parameter was estimated for the symmetrical pairwise between-country migration rate, representing the transmission rate between countries (log-normal prior, mean=-8, standard deviation=2).

## References

1 Eyre DW, Cule ML, Wilson DJ, *et al.* Diverse sources of *C. difficile* infection identified on whole-genome sequencing. *N Engl J Med* 2013; **369**: 1195–205.

2 De Silva D, Peters J, Cole K, *et al.* Whole-genome sequencing to determine transmission of *Neisseria gonorrhoeae*: an observational study. *Lancet Infect Dis* 2016; **16**: 1295–303.

3 Guindon S, Gascuel O. A simple, fast, and accurate algorithm to estimate large phylogenies by maximum likelihood. *Syst Biol* 2003; **52**: 696–704.

4 Didelot X, Wilson DJ. ClonalFrameML: efficient inference of recombination in whole bacterial genomes. *PLoS Comput Biol* 2015; **11**: e1004041.

5 Zerbino DR, Birney E. Velvet: algorithms for de novo short read assembly using de Bruijn graphs. *Genome Res* 2008; **18**: 821–9.

6 Bouckaert R, Heled J, Kühnert D, *et al.* BEAST 2: A Software Platform for Bayesian Evolutionary Analysis. *PLoS Comput Biol* 2014; **10**: e1003537.

7 De Maio N, Wu C-H, O’Reilly KM, Wilson DJ. New Routes to Phylogeography: A Bayesian Structured Coalescent Approximation. *PLOS Genetics* 2015; **11**: e1005421.

8 Babakhani F, Seddon J, Sears P. Comparative microbiological studies of transcription inhibitors fidaxomicin and the rifamycins in Clostridium difficile. *Antimicrob Agents Chemother* 2014; **58**: 2934–7.

9 Eyre DW, Babakhani F, Griffiths D, *et al.* Whole-Genome Sequencing Demonstrates That Fidaxomicin Is Superior to Vancomycin for Preventing Reinfection and Relapse of Infection With Clostridium difficile. *J Infect Dis* 2014; **209**: 1446–51.

10 Leeds JA, Sachdeva M, Mullin S, Barnes SW, Ruzin A. In vitro selection, via serial passage, of Clostridium difficile mutants with reduced susceptibility to fidaxomicin or vancomycin. *J Antimicrob Chemother* 2014; **69**: 41–4.

11 Dingle KE, Didelot X, Quan TP, *et al.* A role for tetracycline selection in the evolution of *Clostridium difficile* PCR-ribotype 078. *bioRxiv* 2018; 262352.

## Supplementary figures


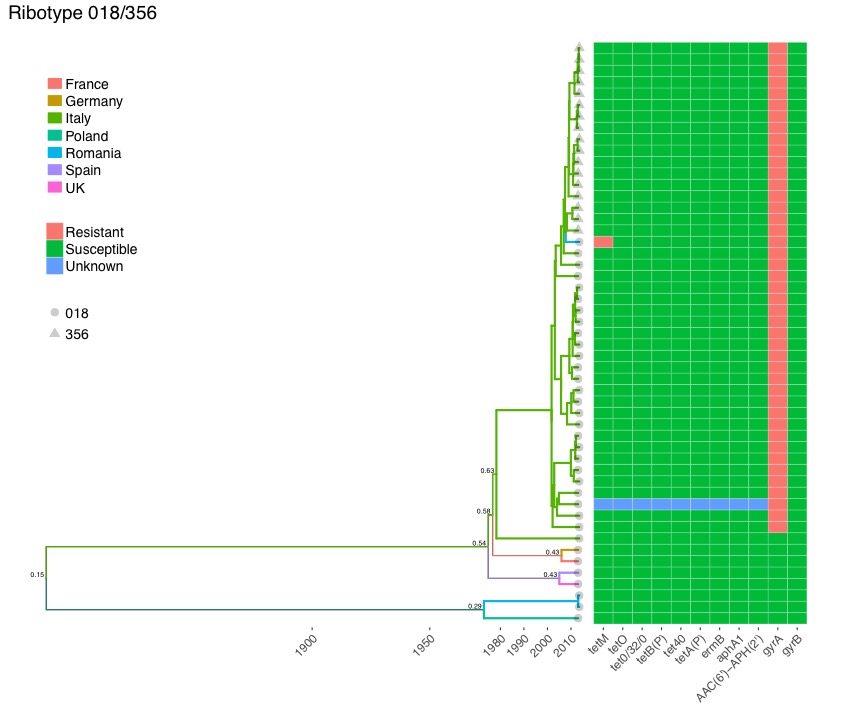


**Figure S1. Combined ribotype-018 and ribotype-356 phylogeny with ancestral country reconstruction and antimicrobial resistance determinants.** The tree tips are annotated with a circle (ribotype-018) or a triangle (ribotype-356).

**Figure S2. Ribotype-001 phylogeny with ancestral country reconstruction and antimicrobial resistance determinants.**

**Figure S3. Ribotype-176 phylogeny with ancestral country reconstruction and antimicrobial resistance determinants.**

**Figure S4. Ribotype-002 phylogeny with ancestral country reconstruction and antimicrobial resistance determinants**

**Figure S5. Ribotype-014 phylogeny with ancestral country reconstruction and antimicrobial resistance determinants.**

**Figure S6. Ribotype-015 phylogeny with ancestral country reconstruction and antimicrobial resistance determinants.**

**Figure S7. Ribotype-020 phylogeny with ancestral country reconstruction and antimicrobial resistance determinants.**

## Supplementary tables

| Accessory gene | Reference sequence (GenBank) | Protein encoded | Antimicrobial resistance phenotype  (predicted) |
| --- | --- | --- | --- |
| *tetM* | NG_048243.1 | Ribosomal Protection Protein | Tetracycline |
| *tetO* | AY394561.1 | Ribosomal Protection Protein | Tetracycline |
| *tetW* | FR838948.1 | Ribosomal Protection Protein | Tetracycline |
| *tet0/32/0* | AJ295238.3 | Ribosomal Protection Protein | Tetracycline |
| *tetB(P)* | NG_048319.1 | Ribosomal Protection Protein | Tetracycline |
| *tet40* | JQ280445.2 | Efflux pump | Tetracycline |
| *tetA(P)* | AB054980.1 | Efflux pump | Tetracycline |
| *tetL* | NG_048203.1 | Efflux pump | Tetracycline |
| *ermB* | HG002387.1 | rRNA adenine N-6-methyltransferase | Macrolide-Lincosamide-Streptogramin B (MLS_B_) antibiotics including clindamycin |
| *aphA1* | M26832.1 | Aminoglycoside 3'-phosphotransferase | Aminoglycoside (streptomycin) |
| AAC(6')-APH(2') | M13771.1 | 6'-N-acetyltransferase and 2"-O-phosphotransferase activities, bifunctional | Most clinically important aminoglycosides |

| Housekeeping gene | Non-synonymous substitution | Protein Modified | Antimicrobial Resistance Phenotype |
| --- | --- | --- | --- |
| *gyrA* | T82I | DNA gyrase subunit A | Fluoroquinolones |
| *gyrB* | D426N | DNA gyrase subunit B | Fluoroquinolones |
| *rpoB* | V1143F, V1143G, V1143L, Q1074K | RNA polymerase subunit β | Fidaxomicin^8,9^ |
| *rpoC* | D237Y | RNA polymerase subunit β’ | Fidaxomicin^8^ |
| CD22120 | Truncation | *marR* homolog | Fidaxomicin^10^ |

**Table S1. Antimicrobial resistance determinants searched for**. Based on resistance determinants previously described by Dingle *et al*^11^**.**

| Ribotype | Mapping | De novo assembly | | |
| --- | --- | --- | --- | --- |
|  | **Proportion of reference genome called** | **N50** | **Number of contigs** | **Megabases of sequence in contigs >1k long** |
| 001 | 0.835 (0.827 - 0.845) | 6627 (4600 - 17802) | 1244 (585 - 1624) | 4.02 (3.87 - 4.13) |
| 002 | 0.829 (0.821 - 0.842) | 8071 (4858 - 28280) | 944 (417 - 1496) | 3.96 (3.81 - 4.09) |
| 014 | 0.839 (0.828 - 0.851) | 6338 (4938 - 11262) | 1261 (752 - 1476) | 3.95 (3.81 - 4.07) |
| 015 | 0.840 (0.831 - 0.846) | 7134 (5071 - 11040) | 1197 (837 - 1497) | 4.16 (4.02 - 4.26) |
| 018 | 0.838 (0.822 - 0.848) | 7246 (5125 - 22680) | 1088 (526 - 1507) | 4.08 (3.95 - 4.19) |
| 020 | 0.843 (0.833 - 0.856) | 6534 (4931 - 25800) | 1168 (554 - 1502) | 3.97 (3.86 - 4.09) |
| 027 | 0.923 (0.911 - 0.934) | 7461 (5151 - 20464) | 1080 (531 - 1460) | 3.94 (3.81 - 4.06) |
| 078 | 0.928 (0.915 - 0.938) | 6198 (4068 - 17192) | 1140 (352 - 1568) | 3.76 (3.57 - 3.90) |
| 106 | 0.828 (0.828 - 0.828) | 4736 (4736 - 4736) | 1610 (1610 - 1610) | 3.78 (3.78 - 3.78) |
| 176 | 0.832 (0.829 - 0.836) | 8008 (5337 - 12079) | 905 (641 - 1039) | 3.98 (3.86 - 4.03) |
| 356 | 0.841 (0.831 - 0.849) | 6635 (5106 - 46375) | 1123 (327 - 1529) | 4.08 (3.90 - 4.12) |

**Table S2. Sequencing quality metrics, median (interquartile-range) by ribotype.** N50 is a weighted median statistic such that 50% of the entire assembly is contained in contigs equal to or larger in length than this value. Samples were mapped to the *C. difficile* 630 reference genome (AM180355.1), with the exception of ribotype-027 which was mapped to the CD196 reference genome (NC_013315.1) and ribotype-078 which was mapped to M120 (FN665653.1).

| Identifier | Ribotype | Collection date | Country |
| --- | --- | --- | --- |
| 4b7b0a87-6283-4b0c-8a06-6baabc112bd7 | 001 | 2013-01-08 | Bulgaria |
| 14503ff2-51cf-4488-8a74-58d8c34e69c7 | 001 | 2013-01-08 | Bulgaria |
| 84c50328-4863-4f97-a509-6505ed55de2c | 001 | 2013-01-10 | Bulgaria |
| f879c402-aa4c-428a-a173-82e5cd256277 | 001 | 2013-01-15 | Bulgaria |
| ca35c6c7-ad70-4f2b-885d-ddd19f69055b | 001 | 2013-07-12 | Bulgaria |
| e43a403a-248b-49ca-926a-ab8688fe2df6 | 001 | 2013-01-14 | Czech Republic |
| cf1aedbd-f655-40f2-8bb0-80a0eee9a2d0 | 001 | 2013-01-15 | Czech Republic |
| 59339567-82ee-414b-ab8d-139596c2e1bd | 001 | 2013-08-10 | Finland |
| 2b8ef645-edb6-4892-a6fc-7277573aedaa | 001 | 2012-12-03 | France |
| d4717931-a4d9-4d9f-8263-0ee0bf7592cc | 001 | 2012-12-06 | France |
| a4b1651e-c807-4339-a7bd-87b5310e408c | 001 | 2013-07-02 | France |
| a79e1932-f0f2-4898-b797-57461d7995da | 001 | 2013-07-03 | France |
| 392f7010-b9b6-45cd-8d28-50b7e062315f | 001 | 2013-01-05 | Germany |
| ef142327-f2fd-44e4-b87b-527d586c22e0 | 001 | 2013-01-06 | Germany |
| 843a58de-a466-43e9-9c0e-960cb52b638c | 001 | 2013-01-07 | Germany |
| 0de0a1cf-4f75-45b1-bf10-95cfa483be5b | 001 | 2013-01-07 | Germany |
| 1f5b0723-4bb0-4cfd-a8a7-64011e902a8d | 001 | 2013-01-08 | Germany |
| 8b081ce1-c3a4-4ee4-bd03-3542e9373ac3 | 001 | 2013-01-08 | Germany |
| 3dff4729-3be6-49ae-8f52-1f16b42e147b | 001 | 2013-01-09 | Germany |
| 3c58ebc1-39a8-475c-8012-4f931bd22e74 | 001 | 2013-01-09 | Germany |
| 466d93de-c1ea-400b-a23a-eb8ac7e82484 | 001 | 2013-01-09 | Germany |
| b526a128-1002-4c34-a41d-51eba89ded84 | 001 | 2013-01-09 | Germany |
| bd70c228-5ddf-499c-8c9c-40c8bf4c1220 | 001 | 2013-01-09 | Germany |
| 336cc750-7417-4571-bc31-cba520d52537 | 001 | 2013-01-09 | Germany |
| 23dac838-5a26-4443-b9e8-0f1992714246 | 001 | 2013-01-10 | Germany |
| 42623497-388a-4769-b953-9273523e31cf | 001 | 2013-01-10 | Germany |
| 3bd58a62-4174-46ce-81f3-fc5a6a9f1e07 | 001 | 2013-01-10 | Germany |
| 26ba0774-0ed4-4ade-a399-f582dc73cc7e | 001 | 2013-01-12 | Germany |
| 20e082e4-5272-4bc9-828a-c90c265312b3 | 001 | 2013-01-14 | Germany |
| 9a8e0ad1-0157-4264-bfef-c718d45b53db | 001 | 2013-01-14 | Germany |
| 03fe8be1-7c6d-476e-96d9-19681c899652 | 001 | 2013-01-14 | Germany |
| 79eacecb-c6b4-4055-b3a5-1a14e794114c | 001 | 2013-01-14 | Germany |
| 9be28881-8ee3-49e6-9b58-3f5244375b08 | 001 | 2013-01-14 | Germany |
| 583ce429-e5fb-4830-af8e-a63dd2078287 | 001 | 2013-01-14 | Germany |
| 01410f45-43da-45d6-b0c5-a2ec0c73b1ea | 001 | 2013-01-15 | Germany |
| 34d1ffbc-ddbe-4817-b5f2-14f457ee9874 | 001 | 2013-01-15 | Germany |
| 51b4d32c-6e21-4f45-9993-7dff819b70b0 | 001 | 2013-01-15 | Germany |
| 6d345b37-f268-4069-bcfe-005e2c94f9a5 | 001 | 2013-01-15 | Germany |
| 4cc5e5d8-dcee-4524-9a6b-67f30c53e506 | 001 | 2013-01-15 | Germany |
| e538f37e-412c-4df5-8d90-de96d98eee29 | 001 | 2013-01-15 | Germany |
| 307685e8-e5c2-4054-909b-c3f83fa881b9 | 001 | 2013-01-15 | Germany |
| d1d31667-0bf9-4910-9793-4aed5519fbf7 | 001 | 2013-01-15 | Germany |
| e7bde746-ba02-4b09-a4ae-fdc0e148cdec | 001 | 2013-01-15 | Germany |
| 41ec0fb5-d020-4864-ac0b-21e9cc53d692 | 001 | 2013-01-16 | Germany |
| 2ee9a963-dfb2-407f-99de-eed42dbe0b2c | 001 | 2013-01-16 | Germany |
| c2ac8014-9d31-4cec-b8d3-9d37da5ea5cc | 001 | 2013-01-16 | Germany |
| 07ac4ba6-16b1-42cb-8667-58eeb000b2a2 | 001 | 2013-01-17 | Germany |
| 4202a7b4-3c88-4d48-bec5-44a6b254ae00 | 001 | 2013-01-17 | Germany |
| f20e76eb-5258-4108-bb6a-ef9a9fc1f489 | 001 | 2013-01-17 | Germany |
| 7fec7648-28ca-48bb-a910-af11604923f7 | 001 | 2013-01-17 | Germany |
| 0f66b338-67bc-4d5e-8d71-7dbe30210654 | 001 | 2013-01-17 | Germany |
| eff5dcc3-f730-4045-bf1e-ecc5802aa3c0 | 001 | 2013-01-17 | Germany |
| a85016fc-f978-44fd-834c-867d9673a62f | 001 | 2013-01-17 | Germany |
| e6ac59f7-6bf6-4a6d-9490-7fa2d6223953 | 001 | 2013-01-18 | Germany |
| bb50b8a9-c26c-4dcc-9c54-cab950c0269c | 001 | 2013-06-28 | Germany |
| f2932da9-5345-4abb-b4bc-ac4acbc80f4e | 001 | 2013-07-01 | Germany |
| 3f6ecfc2-315f-467a-8221-c05a288ce1ce | 001 | 2013-07-01 | Germany |
| 1dc7b0f9-841c-499d-b044-14912da2986c | 001 | 2013-07-01 | Germany |
| d2db9d0c-afbf-4ddb-bdef-f90cefb007d1 | 001 | 2013-07-01 | Germany |
| 0fcd180f-59f7-457c-a067-28879a55a220 | 001 | 2013-07-01 | Germany |
| 2dabba59-7067-473f-83d4-a09e39f52815 | 001 | 2013-07-01 | Germany |
| a73c8bdb-d30b-4ece-8407-65bad64e9cf2 | 001 | 2013-07-01 | Germany |
| f59fa5f8-e692-4727-9f3d-014adb69a653 | 001 | 2013-07-01 | Germany |
| 0ba6a0dd-621f-4af4-9909-20f243f968e6 | 001 | 2013-07-02 | Germany |
| 943b7243-cae0-4ea7-a6f8-1d6219a4b11a | 001 | 2013-07-02 | Germany |
| e0e71a1d-e801-41e6-b65e-8bac8125cd11 | 001 | 2013-07-02 | Germany |
| a6dfcd44-f3cf-471d-aede-3c082461d8f0 | 001 | 2013-07-02 | Germany |
| b4206746-f1bb-4127-935f-d385ff8705ee | 001 | 2013-07-03 | Germany |
| da42c9e3-6596-4c9e-9c33-394bc6b14385 | 001 | 2013-07-04 | Germany |
| 02a08abd-5dcd-4df6-8680-cfda70e84821 | 001 | 2013-07-06 | Germany |
| 9f84182d-7199-453a-8827-35a76d2c91d4 | 001 | 2013-07-07 | Germany |
| 5d870ea4-29ec-4ca0-9587-a91dac968b87 | 001 | 2013-07-07 | Germany |
| cd175877-3677-427c-8681-112fdac40f67 | 001 | 2013-07-08 | Germany |
| fd7bf2b9-f653-46aa-b037-ce1160ddd860 | 001 | 2013-07-08 | Germany |
| 618d7884-a360-4e21-897a-0b59e61f462c | 001 | 2013-07-08 | Germany |
| a0cb61f2-dbb2-4ea4-83ed-eca8f1c482b9 | 001 | 2013-07-08 | Germany |
| 188a7a76-a3ae-4a00-ace6-0087797b7db8 | 001 | 2013-07-08 | Germany |
| d76cf66d-6010-408f-bf20-7740e1f09cd6 | 001 | 2013-07-08 | Germany |
| 6395bdb5-f2e6-4743-a2c4-5660a34065a8 | 001 | 2013-07-08 | Germany |
| bb4c3d87-ab8b-4989-8996-42da143ed7d6 | 001 | 2013-07-09 | Germany |
| 6108543a-5292-4810-9fb2-21ea0e4ff7c3 | 001 | 2013-07-09 | Germany |
| c9a611df-1a80-4f6c-9465-b10e2a532a95 | 001 | 2013-07-09 | Germany |
| 3cd0ed1c-1db8-4c0c-87ee-9269ec3a64bc | 001 | 2013-07-10 | Germany |
| c04c39d1-9a5c-48e2-960a-7fdd27457e1f | 001 | 2013-07-10 | Germany |
| 4f64c90c-7233-4c57-bd17-1bd679cfff23 | 001 | 2013-07-10 | Germany |
| cdb94758-0544-4743-b7b2-2339d4a98ce4 | 001 | 2013-07-11 | Germany |
| a977189c-5bbe-4b61-8c01-b580b590e8d1 | 001 | 2013-07-11 | Germany |
| 78c1c2f1-d69a-4e80-9a0e-5a6b7c5f8ecc | 001 | 2013-07-03 | Hungary |
| 3868f27f-33de-42ec-bf51-b88d2af0df55 | 001 | 2013-07-03 | Hungary |
| f3bcc99f-96dc-470d-b935-827c44ace254 | 001 | 2013-07-03 | Hungary |
| 32baaeb8-9ec5-43c7-a3ec-149b3d6e8d7e | 001 | 2013-07-04 | Hungary |
| d9cbe79c-5f75-4e47-95f6-59b57a29b4af | 001 | 2013-01-08 | Italy |
| a05b15af-0d74-4d20-87ed-4a4c4268b835 | 001 | 2013-07-09 | Italy |
| 63d69dd5-9429-4cda-818b-59cd389b6aee | 001 | 2013-08-18 | Netherlands |
| cb0ea75f-7bbd-4d4d-a160-f688dc36f16a | 001 | 2013-08-20 | Netherlands |
| 67399b93-ca07-4d6a-aea6-1f4125db23ef | 001 | 2013-01-10 | Poland |
| 25d15ab8-f534-4e9b-8151-0ef4b0cd4af9 | 001 | 2013-01-16 | Poland |
| 295c399f-f0ba-4a21-af7a-e01017663293 | 001 | 2013-07-01 | Poland |
| 86a4dcad-a876-4228-a3bf-6ae69a5b2a66 | 001 | 2013-07-10 | Romania |
| 899e11a1-9dac-4b39-970c-c3da99ae4e97 | 001 | 2013-01-15 | Slovakia |
| 27737b77-a906-4d34-9611-19d899e856f8 | 001 | 2013-01-15 | Slovakia |
| f76344dc-b2e8-4109-bd66-4f2100eb22a7 | 001 | 2013-01-15 | Slovakia |
| 3b7c6b43-4750-4753-bbfd-3e2c6a14121a | 001 | 2013-07-02 | Slovakia |
| e894121c-83ce-4bcf-a801-584aa5170215 | 001 | 2013-07-02 | Slovakia |
| 9af7dad7-1579-41da-a35a-fefa04fa741a | 001 | 2013-07-02 | Slovakia |
| 7869d110-cc05-4328-9853-a6f1568def85 | 001 | 2013-07-02 | Slovakia |
| c146feae-ef78-4757-b9bb-ea331deb66db | 001 | 2013-07-02 | Slovakia |
| edacd040-c5c8-4c11-8508-66ff8daebf61 | 001 | 2013-07-02 | Slovakia |
| bd37488c-3ad2-4625-9fe6-841e508a1f3c | 001 | 2013-07-08 | Slovakia |
| 24bc66da-c897-4884-8f45-a99eb0e992b1 | 001 | 2013-07-08 | Slovakia |
| c5a550ef-f6fb-4626-83fb-92c44825e7ca | 001 | 2013-01-15 | Spain |
| 788b9e32-2289-4c60-8635-8d5af7c08a9d | 001 | 2013-01-15 | Spain |
| 4ad0a7c3-384e-450f-9297-07e9fde28c11 | 001 | 2013-01-15 | Spain |
| aec98b63-1a89-4702-98fa-c3d6209d3de5 | 001 | 2013-07-07 | Spain |
| b1000ea0-2cc9-4663-ad8a-0eb367b10043 | 001 | 2013-07-09 | Spain |
| 4b863d32-36dd-4fa5-88c8-a065b85ff336 | 001 | 2013-07-09 | Spain |
| 422908f0-0153-404d-8e39-3b55a68e3e02 | 001 | 2013-01-07 | Sweden |
| 7fdab04c-ddad-495d-99fc-35a1443bb4f0 | 001 | 2013-01-14 | UK |
| 33b36e2a-8740-445c-9c26-1a030eaf074d | 001 | 2013-08-14 | UK |
| 44651c25-e913-4314-ab5e-9b1bbd19bc7e | 002 | 2013-01-08 | Belgium |
| d9b93a7c-fc1f-43c8-90a0-7abfecf33e87 | 002 | 2013-01-13 | Finland |
| d3bea30b-35b6-405d-b688-4b5dc24f54b5 | 002 | 2013-08-13 | Finland |
| a7b86d5e-b173-412b-ac3a-b2a896856a3f | 002 | 2012-12-06 | France |
| 3fa32a3a-d62b-4812-8e85-fd9470a2c8cd | 002 | 2013-07-02 | France |
| e9fd51a4-0bf3-4ded-858d-dee4aae257e2 | 002 | 2013-07-04 | France |
| 695caafc-3608-40b8-84e5-36fb574f241e | 002 | 2013-01-05 | Germany |
| 7f46fef0-0bd4-48ca-8923-14ab441160cd | 002 | 2013-01-09 | Germany |
| 901f2be6-aa38-44a3-a7ca-e5a6a7f76c52 | 002 | 2013-01-12 | Germany |
| 68702961-b374-41d0-bd0d-f3aeb2dfdec0 | 002 | 2013-01-14 | Germany |
| 1b706e7a-3fb2-42c6-bad5-fda3cc06d1db | 002 | 2013-01-14 | Germany |
| eb5fc233-9542-4219-92e2-5d7e42c25f38 | 002 | 2013-01-14 | Germany |
| 1c85cb8e-2bb7-4945-8434-506f905603a4 | 002 | 2013-01-15 | Germany |
| 4f4bc5d9-c995-4235-a52d-742eaa846dad | 002 | 2013-01-16 | Germany |
| 16c86df7-2ae7-4de2-99ac-452f4ffe91c2 | 002 | 2013-01-17 | Germany |
| 1d120f0a-04fa-4f0c-b01e-edf052483a36 | 002 | 2013-07-01 | Germany |
| 356c09ea-fa31-4526-aeea-63c7459abcd9 | 002 | 2013-07-04 | Germany |
| 1cd70cde-adcb-417b-9ddf-9f10d1a8349a | 002 | 2013-07-05 | Germany |
| 5de34a2f-4879-4a09-bd77-d3c5445029e6 | 002 | 2013-07-06 | Germany |
| 5c6d72ea-aec1-47d3-9773-88e91725b4ee | 002 | 2013-07-08 | Germany |
| f228712d-0a14-4de0-be26-78a683c4a0f9 | 002 | 2013-07-08 | Germany |
| 97cf78a8-bb97-4fab-8aa5-c81bd344c9f7 | 002 | 2013-07-08 | Germany |
| 458c1f19-07ff-457c-86a1-3340c664c477 | 002 | 2013-07-08 | Germany |
| 27f2c438-36a1-43ff-b796-9110e15c1e9e | 002 | 2013-07-08 | Germany |
| 4b50f672-aca6-49cf-bec8-d193e34dd5b6 | 002 | 2013-01-07 | Italy |
| e5a8f274-12f7-446c-a1e7-9438b97f3c86 | 002 | 2013-01-07 | Italy |
| 7784dc35-8cf7-4c83-baff-11e7d54f8d84 | 002 | 2013-08-20 | Netherlands |
| 4712c729-b6fa-4e9f-94fe-5f8cc4edc49c | 002 | 2013-01-07 | Poland |
| bb64b507-43cc-410f-93e5-4337ab0126a9 | 002 | 2013-07-03 | Poland |
| d8e1c6e5-9951-4f23-b95b-df3b33807f7e | 002 | 2013-07-10 | Poland |
| 7af1df75-9588-49ec-8310-04c073f0f694 | 002 | 2013-01-10 | Portugal |
| f892d944-6a7c-404d-87f6-f48efc47fa0c | 002 | 2013-07-03 | Portugal |
| 4657991b-9c86-4ff7-9cea-18f9338c5fd3 | 002 | 2013-07-04 | Portugal |
| 12534845-29b3-4e68-a32e-f05ebda8da90 | 002 | 2013-01-08 | Romania |
| bc9e6be5-6079-4899-8e4b-33f0945ab98e | 002 | 2013-07-08 | Romania |
| fcf34c3a-3e7b-48cf-a574-db35f3b7282c | 002 | 2013-01-10 | Sweden |
| 086a58ee-0c8d-4de1-b938-d46658441bce | 002 | 2013-08-16 | Sweden |
| ef761620-4ac5-44d3-8fed-6aa763f0c5b0 | 002 | 2013-01-08 | UK |
| 842313dc-1df0-4425-81e6-894928ba16e5 | 002 | 2013-01-09 | UK |
| 1b59e086-19f5-4bd5-8a24-45209a52009e | 002 | 2013-01-14 | UK |
| 445b2879-a8e4-404d-bc8d-27bf3d8c4a69 | 002 | 2013-01-14 | UK |
| 26179237-e592-407a-8cd3-42d8186f31da | 002 | 2013-08-03 | UK |
| 78a74674-3e3d-43ec-bc50-23db4ec85bcc | 002 | 2013-08-05 | UK |
| 1046d6da-283a-4861-a523-dd7349c8823a | 002 | 2013-08-14 | UK |
| d77bced0-3e85-4ff9-914a-b8eb0c4e2415 | 014 | 2013-01-07 | Czech Republic |
| 59e59615-4b46-4088-9cd0-519bed3288c5 | 014 | 2013-01-16 | Czech Republic |
| 4dbf4979-0d47-43b4-96f9-faae93741d84 | 014 | 2013-07-02 | Czech Republic |
| 918aea29-eb01-4836-8b18-113f69f8cf0f | 014 | 2012-12-03 | France |
| aeaa4ea1-a5d0-4cd9-b93c-2244f995cbdb | 014 | 2012-12-05 | France |
| 0c4122fa-508e-4944-bc49-fe6cb8040ed9 | 014 | 2013-01-15 | France |
| d0059c43-6486-498a-9aa3-6931c237484e | 014 | 2013-07-04 | France |
| 4aa586a1-4561-4c8a-b5e6-5b84e2762621 | 014 | 2013-07-04 | France |
| 7d9e0a01-c1b0-4d89-aff7-ac805db00f87 | 014 | 2013-07-11 | France |
| 1afda199-a0c9-43e6-973d-f031cc712b99 | 014 | 2013-01-07 | Germany |
| 13d8f7de-3c79-4014-b40a-e06ce044e481 | 014 | 2013-01-08 | Germany |
| ef38c68d-db2f-45cd-81fa-bebe3d09ce62 | 014 | 2013-01-09 | Germany |
| 158f8d42-ee38-46b3-8057-2ffa5e13fd0c | 014 | 2013-01-09 | Germany |
| dbb50a49-aef0-40ea-ba6a-efbbddf05e9f | 014 | 2013-01-10 | Germany |
| 6a111c52-2b74-4c3b-a18d-e033524beb44 | 014 | 2013-01-10 | Germany |
| 0c7828b4-a2d2-4c35-b1db-48112195cff1 | 014 | 2013-01-11 | Germany |
| a2f1e821-f0cd-40f6-9b62-4d2eeb28a889 | 014 | 2013-01-12 | Germany |
| cdbc8236-7ae3-49a0-b8b0-e9377bcac8ba | 014 | 2013-01-12 | Germany |
| 02a75819-d25b-4f0f-b287-b8d7aaaa7fbd | 014 | 2013-01-13 | Germany |
| 09800655-2cc3-4f9d-b4b2-a19d5f676723 | 014 | 2013-01-13 | Germany |
| 709aa0c4-fe94-4eab-8318-b51eaf10f4d1 | 014 | 2013-01-14 | Germany |
| 4c8b8c5f-5e78-4ba3-bd4c-ce3ff2f2253e | 014 | 2013-01-14 | Germany |
| 8de24742-c238-4c13-9c44-60b649a0c033 | 014 | 2013-01-15 | Germany |
| c8290e44-8ece-4310-9e46-8352752a1dd1 | 014 | 2013-01-15 | Germany |
| 1f611daf-6ef0-431d-b085-c937890572d3 | 014 | 2013-01-15 | Germany |
| a46c1635-89bf-404b-91c4-7d083931cbcd | 014 | 2013-01-15 | Germany |
| c10b2b4d-2fdf-45cd-8117-4f82ce2e6560 | 014 | 2013-01-15 | Germany |
| 9d882486-4e21-455a-87ff-fd1462470ae1 | 014 | 2013-01-15 | Germany |
| 56a9ad4c-3541-4830-8c44-86fecf0a8508 | 014 | 2013-01-15 | Germany |
| b514bb62-98e6-4118-ace8-87de29e78125 | 014 | 2013-01-15 | Germany |
| 2e42cb8e-53a3-4d59-936b-b16badc66ec9 | 014 | 2013-01-15 | Germany |
| 4b987b82-bf81-43a1-900f-c86df3e861fc | 014 | 2013-01-15 | Germany |
| 375e4a93-cbd5-45d5-ab43-177991753ed1 | 014 | 2013-01-15 | Germany |
| 853dce03-3902-48f6-9871-84323c8e1571 | 014 | 2013-01-16 | Germany |
| 4b0e6ee2-206e-476d-ad9c-61a0b4c5ec4c | 014 | 2013-01-16 | Germany |
| af58f0c8-3d42-4367-9c94-2f264fd365fb | 014 | 2013-01-17 | Germany |
| dbef43d9-5f47-4733-b6cc-e4a5e7df4f16 | 014 | 2013-01-17 | Germany |
| 10a654d0-9ba3-49b2-acb4-f4bad23118f0 | 014 | 2013-01-17 | Germany |
| 1a513901-be69-4bc6-bfff-8250cdf332ae | 014 | 2013-06-28 | Germany |
| edbbe6c2-186e-4f2f-be48-59aea55d1008 | 014 | 2013-07-03 | Germany |
| c44f5830-c0e1-461a-8204-173aac079eab | 014 | 2013-07-03 | Germany |
| c77281f9-711b-48ba-b010-a221efd3fec0 | 014 | 2013-07-04 | Germany |
| f7ddd54b-136b-4c3e-a895-dfd28a0abc62 | 014 | 2013-07-08 | Germany |
| 5ed1c4dd-db08-4278-9374-08dfbda47a4a | 014 | 2013-07-08 | Germany |
| c594acd2-f951-49c9-934b-70f161347ec0 | 014 | 2013-07-09 | Germany |
| 0979f6df-a00e-49d7-91de-6572fd495b17 | 014 | 2013-07-10 | Germany |
| fb85fc8d-c991-4180-a0ba-a3afd268bfa2 | 014 | 2013-07-10 | Germany |
| 622471d7-ab8a-412a-91d4-9999db5b79e2 | 014 | 2013-07-10 | Germany |
| 77849ae4-feee-446a-bee5-108b8a64b1f6 | 014 | 2013-07-11 | Germany |
| 7c21a457-02b1-4f81-9f26-039aa1eb825f | 014 | 2013-07-11 | Germany |
| fa551230-5ae0-43b1-870d-ce790035d9d4 | 014 | 2013-01-08 | Hungary |
| adcdc773-4700-469d-bc7a-bab2269b952c | 014 | 2013-01-09 | Hungary |
| 3fb5ea63-e49b-4431-b544-28d512d03c93 | 014 | 2013-07-03 | Hungary |
| a82f01a1-fb9e-46b1-bb4c-9164eaf5fc47 | 014 | 2013-01-07 | Ireland |
| a715ca91-3511-4612-a15d-0704d987f207 | 014 | 2013-01-07 | Ireland |
| f9b26cf8-cdfe-4f84-9f0f-0f88f8474228 | 014 | 2013-01-09 | Italy |
| cb153bed-001b-44bd-84b1-26389ec4fa0d | 014 | 2013-07-01 | Italy |
| 5aa18f43-9647-470e-b499-5f3e7d486661 | 014 | 2013-07-08 | Italy |
| 45a23d79-53bd-46da-bd03-0e4aa145ce3d | 014 | 2013-07-09 | Italy |
| 9e77234c-cd21-41fd-a965-16430cde9d87 | 014 | 2013-08-20 | Netherlands |
| a7387cce-8443-4bcc-8aa6-3fe96e088108 | 014 | 2013-01-08 | Poland |
| 82e9a78c-8f18-48d2-b666-8fa4cb980938 | 014 | 2013-01-08 | Poland |
| d8728fe9-aed8-4611-a89a-561a3ba0e951 | 014 | 2013-01-08 | Poland |
| 666b6e91-dfbe-4299-9bfd-3afdfde892e2 | 014 | 2013-01-14 | Poland |
| 990bb851-267a-4148-b6f8-e938ed0e29bb | 014 | 2013-01-14 | Poland |
| 8be2e2ad-6a02-4c48-8f74-4a4f8737d598 | 014 | 2013-01-18 | Poland |
| 8357c0a8-2941-406b-be0f-8a16eaca1474 | 014 | 2013-07-01 | Poland |
| e11e375f-b662-428c-b592-7e1043154c72 | 014 | 2013-07-01 | Poland |
| b8a1ef60-b6f8-4168-bd0a-c50260f6b935 | 014 | 2013-07-03 | Poland |
| 8a56a9a3-506e-4f47-912c-a60f4490ad35 | 014 | 2013-07-10 | Poland |
| ede4c81c-94c5-4ebe-a9e5-812824031858 | 014 | 2013-07-01 | Romania |
| 750f23bd-0087-4d8c-92c5-02e94686ce5e | 014 | 2013-07-02 | Slovakia |
| df670f1f-675c-42da-8544-255ed72783c5 | 014 | 2013-07-09 | Spain |
| c5b7dbb4-a96e-4c67-849f-f432256ce1de | 014 | 2013-01-14 | Sweden |
| 61d6c3fe-1e1c-4683-ad2e-5e2c54cae9a9 | 014 | 2013-08-16 | Sweden |
| 6e0e9c0b-470a-4217-9957-e34fac9c4aa0 | 014 | 2013-01-16 | UK |
| de4fbb4f-b018-4b36-a57e-f5cbc8955294 | 014 | 2013-08-07 | UK |
| 18573737-10c8-4284-ae92-70af849376e5 | 014 | 2013-08-13 | UK |
| d42d479d-4843-45b4-aff6-0a74b5f78b44 | 014 | 2013-08-13 | UK |
| ebbbae47-68f4-486e-8a77-6fd39f6998ed | 014 | 2013-08-15 | UK |
| 283645d7-4ecb-4122-8c12-a87c1eb4c36b | 015 | 2013-07-03 | Bulgaria |
| ead84b61-2ee7-47d1-baf1-807cf4df3812 | 015 | 2013-07-12 | Bulgaria |
| ca7c7a88-07b9-467a-8d09-6f6bb6328410 | 015 | 2012-12-06 | France |
| 0f7d2fa3-b3b3-4e36-a95e-97a4ea890b35 | 015 | 2012-12-06 | France |
| 2da4015c-5ede-44ac-b951-4e45903d4d15 | 015 | 2013-07-01 | France |
| c9d82f49-66c0-4c3d-956f-16cfd0bf767a | 015 | 2013-07-02 | France |
| 692d49b6-89fc-4a1b-a639-1a594c7f1151 | 015 | 2013-07-05 | France |
| ebd7d537-5fb3-4c63-94e4-c9f91d8f391d | 015 | 2013-01-05 | Germany |
| 0def3f51-a668-48d9-9bdc-93b96ff2af13 | 015 | 2013-01-05 | Germany |
| 82d12bb8-09bb-4a6e-9ad6-d3fc29c29cc8 | 015 | 2013-01-06 | Germany |
| 3544fb09-e25e-4664-bde3-79dee233e7dd | 015 | 2013-01-15 | Germany |
| 75238e17-6a7b-4446-9945-0d1298b02cfc | 015 | 2013-01-15 | Germany |
| 169c9a31-dde8-4f14-a1a3-bbdf7d311549 | 015 | 2013-01-15 | Germany |
| 2132cdbe-c726-4d71-833e-1858fe111056 | 015 | 2013-07-08 | Germany |
| 00f39c39-6e0f-49ca-b73b-711c5ad2e713 | 015 | 2013-07-09 | Germany |
| 8dfc7985-bf6c-49e8-96a0-6c7c070510a6 | 015 | 2013-07-10 | Germany |
| b1b79218-efb0-472e-bd6b-2b4cc15e1b6b | 015 | 2013-07-10 | Germany |
| baa287b7-0db8-48ab-a182-aee415081e87 | 015 | 2013-07-10 | Germany |
| 82ae77a0-45da-4b22-89bb-ac5addce6e9d | 015 | 2013-07-10 | Germany |
| 3dbe9a55-9775-4173-adeb-ea6f7b82264c | 015 | 2013-08-06 | Ireland |
| e5395a1d-2407-474a-98e3-3f0a60f37ed7 | 015 | 2013-08-19 | Netherlands |
| d4ecdf8f-17cd-4dd5-a996-aa7f01dd8cba | 015 | 2013-07-09 | Romania |
| 1420a50d-2a5e-45e1-8511-92d214947426 | 015 | 2013-01-09 | UK |
| 00f8a018-4b21-4f25-8674-74a32b49838f | 015 | 2013-01-16 | UK |
| dd993651-e43b-41d7-a57a-f67cb8b5d560 | 015 | 2013-01-17 | UK |
| f67ee435-de03-4041-a402-5b7b3e9d6c15 | 015 | 2013-01-18 | UK |
| 159626c5-7875-479f-a42e-7e6022bf6a97 | 015 | 2013-08-05 | UK |
| 8c5eaffb-8945-4da6-95bd-d49dd2c14b03 | 015 | 2013-08-05 | UK |
| aa390482-c3d6-4c70-a10e-a985f05b73e5 | 015 | 2013-08-16 | UK |
| 16ee2374-d40b-430e-b5aa-9db5c238d5e3 | 018 | 2012-12-07 | France |
| 4d96c7d2-0ad9-4658-b46d-bc17f176e00e | 018 | 2013-01-17 | Germany |
| d2d977c9-c683-4735-a98c-8dcec9f3798f | 018 | 2013-01-07 | Italy |
| cf0e0bf0-6120-42e0-87ef-d72e8c8579c3 | 018 | 2013-01-07 | Italy |
| 2f0cdb3a-db75-4e89-b404-3b037539308b | 018 | 2013-01-07 | Italy |
| 89005d2a-7bb3-4674-b019-c37aab6d3577 | 018 | 2013-01-08 | Italy |
| 28d2f2da-fdfc-4deb-b509-f0ebfc7f1245 | 018 | 2013-01-08 | Italy |
| c5d7d7bd-cd59-4fa7-bd99-20b186e9eb4d | 018 | 2013-01-14 | Italy |
| 43cafbc5-b244-4633-a915-9086bffa0dbd | 018 | 2013-01-14 | Italy |
| d66e06df-ff53-4781-858d-be7c41e7b826 | 018 | 2013-01-14 | Italy |
| d23af771-3de4-45ab-8582-69fc0862815e | 018 | 2013-01-14 | Italy |
| 3de011fd-72cc-41c3-acc1-7cee377b7705 | 018 | 2013-01-15 | Italy |
| 18a54634-738a-4a62-bcff-97faaca5c1f7 | 018 | 2013-01-15 | Italy |
| 2e4b89e8-d74d-4cfd-9435-887841579d06 | 018 | 2013-01-15 | Italy |
| dd4acc72-9379-4f27-b406-bb6d5c6cf758 | 018 | 2013-07-01 | Italy |
| 30376183-ad2c-4faa-b56b-123e92e6416b | 018 | 2013-07-01 | Italy |
| ec2c3742-114a-4c8a-8115-d4fe6c9570b8 | 018 | 2013-07-01 | Italy |
| 9a049732-f9b1-4038-9b18-1f7cf0465b20 | 018 | 2013-07-01 | Italy |
| eca283c6-d52e-485f-b1f2-a50e2535913b | 018 | 2013-07-01 | Italy |
| 7b655891-8784-4537-8438-646d043e60b2 | 018 | 2013-07-02 | Italy |
| 82aa57a7-b034-4d79-a55e-bd14d091cbcb | 018 | 2013-07-02 | Italy |
| 83a90971-0e1e-4b8e-9561-cfe700b4a49f | 018 | 2013-07-02 | Italy |
| b5ca7a2d-2502-4cc6-a64c-30690330f29d | 018 | 2013-07-03 | Italy |
| bd71a382-0471-400b-a4a9-4a80b6edfbac | 018 | 2013-07-08 | Italy |
| 85cc0398-8a0e-46d4-9089-387371319242 | 018 | 2013-07-08 | Italy |
| cbc5d3bc-802f-4f33-bb3b-d4b34ba1b797 | 018 | 2013-07-08 | Italy |
| 920c2e4b-9145-46e2-819c-bc6e896ae060 | 018 | 2013-07-08 | Italy |
| 82851927-9adc-4830-aba0-b33ec420ed6b | 018 | 2013-07-08 | Italy |
| 6785875a-f567-40c0-bc28-8e7b006f95a9 | 018 | 2013-01-08 | Poland |
| cf8465bf-08bf-4f1c-9820-7dd64dc9f6ec | 018 | 2013-07-08 | Romania |
| 9c346472-f3ea-4ca5-aebc-a9da9bb258fc | 018 | 2013-07-09 | Romania |
| 99e2e339-44d0-4c46-aef7-eac83c7202dc | 018 | 2013-07-09 | Romania |
| 9b35b965-bb57-4995-8382-c02450ad15fd | 018 | 2013-01-15 | Spain |
| 0f0d55ad-04ad-4357-a6fa-a7f2606fade2 | 018 | 2013-01-11 | UK |
| ca1df782-0a27-423b-add6-f8c2d993842c | 020 | 2013-01-14 | Belgium |
| 7e71316e-4a59-47bf-b2c1-a1a0842543ec | 020 | 2013-01-14 | Belgium |
| 997963f5-88ef-46f0-93d1-39dc420b5a57 | 020 | 2013-07-10 | Belgium |
| 06ca85c7-8e4e-436f-bac2-fe64e5ac7b81 | 020 | 2013-01-14 | Czech Republic |
| a230f35a-9c33-47ab-8bf8-551cf217ef1f | 020 | 2013-07-02 | Czech Republic |
| 9952ff5a-c3f5-49bc-af62-ae599cda8524 | 020 | 2013-07-08 | Czech Republic |
| e607d038-4490-4b88-829e-367aa5a8c350 | 020 | 2012-12-03 | France |
| 42028b8f-5efd-468a-ba86-c36b094da394 | 020 | 2012-12-04 | France |
| b5865284-149a-45a0-9e38-6d3b68997f73 | 020 | 2013-01-10 | France |
| 245d5647-395a-4838-af3d-7a2f87fca144 | 020 | 2013-01-15 | France |
| 033e560f-ac98-42b8-8e47-208765db1640 | 020 | 2013-07-03 | France |
| 266087f8-12c4-4bf9-821e-c10a3702ad9c | 020 | 2013-07-03 | France |
| ac30e74a-3504-4383-95bd-5d2e4bc95110 | 020 | 2013-01-07 | Germany |
| c438f4e6-212b-43ed-a220-b734a7ae0de4 | 020 | 2013-01-09 | Germany |
| 6e57f5be-9ffa-484d-aa8d-39f5e5881275 | 020 | 2013-01-10 | Germany |
| 8c5113e3-d3e7-42a3-8a02-6af7d0c02fc4 | 020 | 2013-01-14 | Germany |
| 92675b39-9a7a-4241-9918-8a850a64ce73 | 020 | 2013-07-01 | Germany |
| e3cfcef7-5c79-4862-9c8c-b4adb8d39a94 | 020 | 2013-07-01 | Germany |
| 4daaf9fb-ae24-4feb-90c2-1a635de3a0e7 | 020 | 2013-07-01 | Germany |
| afdbfd53-062a-4491-89f6-02bbfb8b5546 | 020 | 2013-07-01 | Germany |
| 4b2de583-b87d-40e6-9b5c-5d45c3d38477 | 020 | 2013-07-05 | Germany |
| f1f58c40-40bf-471d-bf17-3f0ca67af62b | 020 | 2013-07-08 | Germany |
| 86ae0d42-3b81-4dda-ad87-89e4e4ab1054 | 020 | 2013-07-10 | Germany |
| 399645b8-1112-4bcd-93cc-5a28a6b9e7be | 020 | 2013-01-15 | Italy |
| cdcb81e0-f406-4ca7-91b2-600d65270fea | 020 | 2013-07-01 | Italy |
| 7ac75ef3-523e-47a5-b749-6660fea55938 | 020 | 2013-07-01 | Italy |
| fe9fe22c-0afe-4d46-8d84-1f566dc7e63d | 020 | 2013-07-02 | Italy |
| 99a4feea-c6af-48cc-93cd-877afb2e62a9 | 020 | 2013-01-10 | Portugal |
| 325b067b-60fd-4c91-b93a-ea2f135013dc | 020 | 2013-07-11 | Portugal |
| 140fb5d9-9aa8-48f0-a361-4db50f65e428 | 020 | 2013-01-08 | Romania |
| 80a3b416-1fb5-47bb-ad52-f340a468637c | 020 | 2013-07-12 | Romania |
| d8710335-8a5e-40b8-8cc3-d47a5a1a42df | 020 | 2013-01-10 | Sweden |
| 5edaf595-9bdd-4277-8dff-af4b452a7bcf | 020 | 2013-01-21 | UK |
| e24c30bf-0aa4-4c8d-a23b-1f82ed1636ed | 027 | 2013-01-15 | Austria |
| 1a7587a7-8941-4149-81b3-c78c99a39c96 | 027 | 2013-07-02 | Austria |
| 6ed29386-18e1-4586-bf9b-c277468197a5 | 027 | 2013-01-15 | Belgium |
| 16d035da-8c2b-4d5b-a0f8-ea9ce071b789 | 027 | 2013-01-15 | Belgium |
| a07e7823-27ab-4e44-a73c-592e6a994466 | 027 | 2012-12-04 | France |
| 16fb4312-da62-4f7a-8efa-f0e7e8286b87 | 027 | 2013-07-08 | France |
| 1a776d82-5d2b-4d39-be43-8c7ff138aadc | 027 | 2013-01-05 | Germany |
| ce8d4dff-3ea4-4a34-9173-d59baf2297b8 | 027 | 2013-01-06 | Germany |
| 71e4628a-e228-4dd9-bee0-309045ce83fc | 027 | 2013-01-06 | Germany |
| 60926fe7-81c1-4944-b4c0-ea329e43601c | 027 | 2013-01-09 | Germany |
| 4eef0038-1bcf-47e2-9d97-8d0391b9465d | 027 | 2013-01-09 | Germany |
| d20dc835-71dd-4e3a-8f39-9973d715f77f | 027 | 2013-01-14 | Germany |
| 5659b6ee-1619-486a-8046-cd0fa79809a3 | 027 | 2013-01-14 | Germany |
| d8ab45b6-b259-4536-aebe-b7ec795b39a7 | 027 | 2013-01-14 | Germany |
| 71cadc1f-4da1-49d5-9569-c7f9096c10fb | 027 | 2013-01-14 | Germany |
| 17b30d89-6570-4ed7-98a5-a21e50cd120a | 027 | 2013-01-14 | Germany |
| 0b60a3d4-8cc6-464b-a0f6-4034f842ee78 | 027 | 2013-01-15 | Germany |
| 9ae6e032-b7a5-4766-81ca-14562f853254 | 027 | 2013-01-15 | Germany |
| 0c1b8825-c95a-4ad5-b452-761baacfd3ee | 027 | 2013-01-15 | Germany |
| f4b77446-0e84-4f8c-95c9-6687753e59f7 | 027 | 2013-01-15 | Germany |
| e6836a0f-ee95-4542-a6ed-7617087dcc8b | 027 | 2013-01-15 | Germany |
| 1cd3be23-9d73-4954-8b00-9a9f0a940d2b | 027 | 2013-01-15 | Germany |
| ae547871-b33c-4c02-9f14-406536069d57 | 027 | 2013-01-15 | Germany |
| 8d9840f7-5510-4162-98fa-1dbaf465cb2e | 027 | 2013-01-15 | Germany |
| d5967bd7-3556-4e3e-a6ea-5ebbc363bb7c | 027 | 2013-01-15 | Germany |
| 41501b63-1cf2-49d9-bc03-03635bd53a76 | 027 | 2013-01-15 | Germany |
| fec5e70b-49f4-44c8-8c36-7c12a9dd3b3a | 027 | 2013-01-15 | Germany |
| 85833728-be79-4f73-8470-74c5cc343b24 | 027 | 2013-01-15 | Germany |
| df9cd79b-d32f-4c3f-87c1-38a5da31c6d7 | 027 | 2013-01-15 | Germany |
| 9f3a6c12-73f2-40d0-9472-3610374396fa | 027 | 2013-01-17 | Germany |
| 0d7d75d4-48df-4a6f-a185-817fb91b22e5 | 027 | 2013-01-17 | Germany |
| c1c854b1-ba21-44a6-8196-e855ba3d6da7 | 027 | 2013-01-17 | Germany |
| f82f2ccc-e7a8-4f64-bafe-3e9036e90d25 | 027 | 2013-01-17 | Germany |
| 7b6aa0e7-9411-4010-a80b-c0ff91f1f234 | 027 | 2013-01-17 | Germany |
| 020f521f-89c9-4c91-83fb-a05d06e64876 | 027 | 2013-01-17 | Germany |
| 1fa8c367-cc48-4fe2-b55a-66b2f87249f2 | 027 | 2013-01-17 | Germany |
| 1359ae96-fb6f-4c49-b25b-71984581e4b9 | 027 | 2013-01-17 | Germany |
| feef86f2-577a-46fa-b9bb-ee04dedd3d87 | 027 | 2013-01-17 | Germany |
| f063c71b-7f91-4617-b34e-c1f2683c7312 | 027 | 2013-01-17 | Germany |
| 501b23f5-5cf7-4fa3-9364-57c61cbee4cf | 027 | 2013-01-17 | Germany |
| c22e5c32-9ff5-4f0b-8cba-944b2861766d | 027 | 2013-01-17 | Germany |
| 478bff50-0545-4215-83a9-169ae65fed79 | 027 | 2013-01-17 | Germany |
| 6fcf4d7a-75d8-4f29-bee5-2663a2263209 | 027 | 2013-01-17 | Germany |
| dbbfb8c8-8dea-4906-9c58-e6d2f201030c | 027 | 2013-01-17 | Germany |
| 6e41e8b6-0e6d-4165-8bd3-e2f3ce7e716d | 027 | 2013-01-17 | Germany |
| a19205e3-dd3e-4ad7-9bd5-b6a9f5c842b0 | 027 | 2013-01-17 | Germany |
| 87be880c-db9b-46b1-a6a3-039667c3ecb5 | 027 | 2013-01-17 | Germany |
| 279e5578-90ec-4762-a36e-50a53cc3b093 | 027 | 2013-01-18 | Germany |
| 189ba052-799a-40ba-b10c-33ba304fab04 | 027 | 2013-07-01 | Germany |
| 51799c39-a69a-41ab-a8cc-400795e97b59 | 027 | 2013-07-01 | Germany |
| 5a4f6ef3-c3d4-44c4-be52-8e711a081327 | 027 | 2013-07-01 | Germany |
| c3d5e795-8603-4de6-8f3f-5f035b689464 | 027 | 2013-07-01 | Germany |
| 90ba3dbb-2313-409b-acb9-2cbff2537a02 | 027 | 2013-07-01 | Germany |
| 3276a83f-b182-444c-9067-d27080d16093 | 027 | 2013-07-01 | Germany |
| 6c5cfe7d-4270-4261-b2d0-1d885fde3b57 | 027 | 2013-07-01 | Germany |
| 700914be-2e8e-4a1c-875d-b587152dd621 | 027 | 2013-07-01 | Germany |
| 284cdc04-b6fb-4702-872c-f9ab3b5c8020 | 027 | 2013-07-02 | Germany |
| cf63dc97-723c-47dc-a09b-392a4f2585c2 | 027 | 2013-07-04 | Germany |
| fded7932-8b64-4c05-b38f-04823e2142a5 | 027 | 2013-07-04 | Germany |
| 81fe291d-09b6-4af2-8fcd-32f8348b0414 | 027 | 2013-07-04 | Germany |
| 97072105-75f0-4ad6-82b9-4d5637760827 | 027 | 2013-07-04 | Germany |
| 81587091-02cd-4e8d-97ce-bcae04614e3e | 027 | 2013-07-04 | Germany |
| 7bfc156b-b273-496e-a967-dbbd8c90a974 | 027 | 2013-07-05 | Germany |
| fb3ebbdb-d824-47a1-bd97-119f54f407f1 | 027 | 2013-07-05 | Germany |
| 1de2328f-558e-4fa8-8504-9a32ea6c968f | 027 | 2013-07-08 | Germany |
| c94e719a-d040-435b-8521-8746155ce33d | 027 | 2013-07-08 | Germany |
| 5dfc596c-313f-4029-8d7a-8d79031a6e47 | 027 | 2013-07-08 | Germany |
| ed2d12e9-8e1a-4ecd-96f4-d0e239ca86c8 | 027 | 2013-07-08 | Germany |
| c59e1f3b-057b-415c-aa45-00b5d1a5394d | 027 | 2013-07-08 | Germany |
| b9693c3d-6f55-42bd-a7ba-0cc5bc741b37 | 027 | 2013-07-08 | Germany |
| 9ba51ef8-ffdb-4390-98e0-d525931e1d6f | 027 | 2013-07-08 | Germany |
| fe15b094-e0da-451c-b159-efc34c6edd3f | 027 | 2013-07-08 | Germany |
| dd6eb510-1ea7-4819-b2ba-83c58f0ea488 | 027 | 2013-07-08 | Germany |
| 272e0878-06d5-4633-95cd-ec02fc1e02aa | 027 | 2013-07-09 | Germany |
| 9d5bde4b-82d8-416c-a4d4-44cee6d26160 | 027 | 2013-07-09 | Germany |
| dd286d47-519b-4162-9993-1908413f0adb | 027 | 2013-07-09 | Germany |
| de2bc4a7-4daf-4729-9c29-0fe6d63b5645 | 027 | 2013-07-09 | Germany |
| d21ddc9c-f962-4410-9be8-55e20da464ab | 027 | 2013-07-09 | Germany |
| a684adb9-1f3f-49c0-adf0-098ff2c3b81b | 027 | 2013-07-09 | Germany |
| b515a5ee-3999-4fcb-be59-8208d185509b | 027 | 2013-07-09 | Germany |
| 2f08c81a-026c-47aa-9a35-fa60d9d92d9e | 027 | 2013-07-10 | Germany |
| ac48432c-3081-4784-8a3f-fc8259700fc4 | 027 | 2013-07-10 | Germany |
| 224d34b0-0c82-4587-9edc-9344829cbefe | 027 | 2013-07-10 | Germany |
| b5b52508-9970-4b91-9100-b2ba1cc2bc85 | 027 | 2013-07-10 | Germany |
| 823e80d0-e861-4ac0-8c25-27dc69b70858 | 027 | 2013-07-10 | Germany |
| d6db8a3f-5e82-4cb9-848f-34c1ef7bb7cd | 027 | 2013-07-10 | Germany |
| a0c17367-3c3e-4e41-a162-7966a46d5880 | 027 | 2013-07-10 | Germany |
| 0eea773f-24ad-4608-8900-3afdbee65e3a | 027 | 2013-07-10 | Germany |
| b3143377-7918-4b66-bdae-0961565aa2ab | 027 | 2013-07-10 | Germany |
| 3bfbd855-7f94-48e4-a416-3e317f868d57 | 027 | 2013-07-10 | Germany |
| 20dfabfd-d236-4738-8892-b17eccc38a2e | 027 | 2013-07-10 | Germany |
| 2fcb73f2-301e-413c-8422-6fae59299e24 | 027 | 2013-07-10 | Germany |
| 972bacc4-bece-4d23-8e28-8fbcacb47859 | 027 | 2013-07-10 | Germany |
| 150a913a-ce78-4f5b-8e76-68d4a51f24bb | 027 | 2013-07-10 | Germany |
| f63df362-7378-496e-8ada-72578c1e4580 | 027 | 2013-07-10 | Germany |
| 67429dab-e352-4d5d-a5ba-f614e6c678cb | 027 | 2013-07-10 | Germany |
| ccd9a2f1-1a32-4993-a102-b05a8565885f | 027 | 2013-07-10 | Germany |
| 53a4b5d1-1bc3-4c7f-830b-e063ff9ede18 | 027 | 2013-07-10 | Germany |
| 67a036f0-2b67-49dd-8d7e-465fe2e8f027 | 027 | 2013-07-10 | Germany |
| 19d1850f-c410-4101-bd30-03e90e2e42fc | 027 | 2013-07-10 | Germany |
| 78acc10f-d065-47b1-9655-be7f3cb4cb5b | 027 | 2013-07-10 | Germany |
| 888bb21c-7e9e-4a90-acec-381022642457 | 027 | 2013-07-10 | Germany |
| 76b4c2aa-49af-4584-a61c-7096171e5db0 | 027 | 2013-01-08 | Hungary |
| 2f70bc88-c05d-4e8a-b065-b223a873aca6 | 027 | 2013-01-08 | Hungary |
| 40c4bc19-6c76-4dc2-9190-3a865de9ad04 | 027 | 2013-01-08 | Hungary |
| dbdfde2f-ddea-4978-9584-fe63c1ce88b2 | 027 | 2013-01-08 | Hungary |
| 43ffad0a-aa9c-4d25-b91a-3c3fe26f2de1 | 027 | 2013-01-08 | Hungary |
| 8c054e93-29b3-4ab1-bb18-9aefadd09f16 | 027 | 2013-01-08 | Hungary |
| c84d0920-dd7a-40a6-bcf9-5ccedb30a392 | 027 | 2013-01-09 | Hungary |
| 186963f0-f242-4983-b839-189954d43f96 | 027 | 2013-01-11 | Hungary |
| 4905d6bb-db6d-43a2-a168-089acd4f7430 | 027 | 2013-01-11 | Hungary |
| 53ed1608-7caa-4cea-9000-5ba52159f638 | 027 | 2013-01-11 | Hungary |
| 9604578b-9901-4d2e-b349-c3e8feea2f34 | 027 | 2013-01-11 | Hungary |
| 6cfb1b7a-3ce4-4a93-a2da-341cf2e367a2 | 027 | 2013-01-11 | Hungary |
| d5268a73-87f0-4aac-a8fa-9d33a306c2b9 | 027 | 2013-01-14 | Hungary |
| 7d62436a-f904-4988-8a33-11a0222e31f6 | 027 | 2013-07-02 | Hungary |
| edc6983d-d6e8-4ff3-9fff-5f09ed80f338 | 027 | 2013-07-02 | Hungary |
| 9f33f80f-b72c-4eac-810e-b9252c46ffa3 | 027 | 2013-07-03 | Hungary |
| be9da5d9-f914-4db4-a6a1-e45d7a8ae331 | 027 | 2013-07-03 | Hungary |
| ad9580f6-de20-43be-9b29-62af67e0eeca | 027 | 2013-07-03 | Hungary |
| 7930d273-5c1b-4d66-80e9-33d866826ac9 | 027 | 2013-07-03 | Hungary |
| 5df30b8c-0d50-43ea-9af9-de7412e03070 | 027 | 2013-07-03 | Hungary |
| bc8aff73-e7d7-4d33-acc3-7097f06f67c9 | 027 | 2013-07-03 | Hungary |
| 79b2bd8e-bb35-4596-99ae-2cc89afd8e8c | 027 | 2013-07-03 | Hungary |
| 885ca469-48c2-4d05-a5e4-954e153a31e8 | 027 | 2013-07-04 | Hungary |
| 139fe94a-773b-46ee-932f-e76cb36eb54f | 027 | 2013-07-04 | Hungary |
| 09789ff8-f78d-4c02-bd39-b51fc9c945ee | 027 | 2013-07-04 | Hungary |
| 54239829-d0e3-485b-8ecf-04efb5b3a32c | 027 | 2013-07-04 | Hungary |
| 530d33d7-9d40-44f4-bbfe-09050baa3acf | 027 | 2013-07-04 | Hungary |
| 56ff2b5a-0219-4bc5-8de4-7e2ba0df66f1 | 027 | 2013-07-04 | Hungary |
| f1959f18-9a54-4ab4-b198-db26a027ceb9 | 027 | 2013-07-05 | Hungary |
| a539c13c-1a72-4732-ba2a-e9dadcc8bf7e | 027 | 2013-07-09 | Hungary |
| b52bfe07-aa1b-4c02-820e-3c5bc1d22ec4 | 027 | 2013-07-09 | Hungary |
| 641467d7-6d67-40e0-ac8c-9b5868fce828 | 027 | 2013-07-10 | Hungary |
| 453d7722-5fc1-48ce-88fa-035b53e4bb5f | 027 | 2013-07-10 | Hungary |
| 95811654-ba09-4fc4-8217-78aa300c82da | 027 | 2013-07-10 | Hungary |
| d6f2db44-5f86-4a8c-a928-4322a42ee4c2 | 027 | 2013-07-10 | Hungary |
| 59b3007c-16f6-44ba-86ce-c14b3d22f410 | 027 | 2013-07-10 | Hungary |
| 8582cf89-91d1-4555-88ac-badf01928936 | 027 | 2013-07-10 | Hungary |
| ba4d2d35-3244-44a8-8618-438971cd428d | 027 | 2013-07-10 | Hungary |
| de278092-b5e4-435a-a16d-779ee75e7e08 | 027 | 2013-07-10 | Hungary |
| eea6db2e-dd37-4d31-9f49-b81d9718cdd6 | 027 | 2013-01-09 | Italy |
| 9c950502-1025-457e-ad1f-dc102910ee40 | 027 | 2013-01-09 | Italy |
| e3b952ba-882e-42a0-8370-5ee2d847cba6 | 027 | 2013-01-09 | Italy |
| 6a748f31-4c1c-4275-8637-372046f09b90 | 027 | 2013-01-09 | Italy |
| 056a2f99-0ae0-4273-9a72-625e17fc4185 | 027 | 2013-07-01 | Italy |
| 7306522e-7696-41f4-b51b-d72dbb61ee09 | 027 | 2013-07-01 | Italy |
| 935d0756-629d-4a8b-ac8c-3c8d1e4c7f22 | 027 | 2013-07-01 | Italy |
| ae678eac-414a-4d3b-9b1a-20ee19b49752 | 027 | 2013-07-01 | Italy |
| 0e57ab27-6dcc-4e3f-87fd-8d7b5639b4d9 | 027 | 2013-07-01 | Italy |
| 32ae8086-2a59-4245-9906-e1b8e61a6e14 | 027 | 2013-07-09 | Italy |
| 8c47bef5-38dc-4342-a6b2-e3c627fdf65c | 027 | 2013-01-07 | Poland |
| acd68ddd-5f41-4dfd-9889-1bb6c12c93a4 | 027 | 2013-01-07 | Poland |
| 02874d2b-7c01-4c3a-a8e9-c8f26c7fb13d | 027 | 2013-01-07 | Poland |
| 2683e05d-efcf-4e2f-bcb6-81da7e82cf9d | 027 | 2013-01-08 | Poland |
| 283b7743-a418-4b68-9317-d46034bf1d61 | 027 | 2013-01-08 | Poland |
| 7a2dfb2c-c5e2-4017-9acd-916ca6dc9768 | 027 | 2013-01-10 | Poland |
| 9222f029-74d5-48dd-8c84-99a94276749a | 027 | 2013-01-10 | Poland |
| 479e9a6e-75d4-418e-a6fb-088ca7e850d1 | 027 | 2013-01-10 | Poland |
| c1306d30-c09f-4e13-9320-24205e218c27 | 027 | 2013-01-11 | Poland |
| ffce4adc-2e04-461c-aa43-676cc103ed3c | 027 | 2013-01-14 | Poland |
| 5bfa40ae-daa3-4f20-8fcd-8e71650a5308 | 027 | 2013-01-15 | Poland |
| e56e9549-3d2e-498b-9a70-6de383fcecb8 | 027 | 2013-01-16 | Poland |
| 3d0fd053-a822-4bd4-9ea3-6de9d5ee484c | 027 | 2013-01-17 | Poland |
| aea28cdb-b300-4bae-aa26-0cf6bb204698 | 027 | 2013-01-17 | Poland |
| 945e9069-faae-4ca7-af2d-14f33f6b29fa | 027 | 2013-01-17 | Poland |
| e5255d85-13ba-44bb-ba58-321172f39f44 | 027 | 2013-01-17 | Poland |
| 08586dd3-ac2d-4cb3-a3af-ccaec2aef42b | 027 | 2013-01-18 | Poland |
| 08fe8bcd-47a4-438d-881c-b0582543025c | 027 | 2013-07-02 | Poland |
| 7764c29b-6add-4fed-8da1-574ff83b0f5f | 027 | 2013-07-02 | Poland |
| a3dc4fad-4045-4c0b-8d3d-70e9322c9e26 | 027 | 2013-07-02 | Poland |
| a1934466-fc3f-48cb-be94-eb6ad2d78048 | 027 | 2013-07-02 | Poland |
| 59ca70e6-4b87-4785-b827-bffff9a504f4 | 027 | 2013-07-03 | Poland |
| 4a8937f1-beb9-4442-8b71-1470d1490b17 | 027 | 2013-07-03 | Poland |
| b1668278-ad57-4070-a6e8-66b2efa9e5de | 027 | 2013-07-03 | Poland |
| 75557a4e-bfe3-48f9-8493-bc3b91db56b7 | 027 | 2013-07-03 | Poland |
| e3d1450f-f186-4b02-94fb-303a55ceb082 | 027 | 2013-07-03 | Poland |
| faff421f-7b2e-4be4-aee3-6baf0f2f74b7 | 027 | 2013-07-03 | Poland |
| 55bd8143-95ec-494c-8503-9a197ebc42c0 | 027 | 2013-07-08 | Poland |
| 8013ec9a-634d-499c-b777-cc7a805498d7 | 027 | 2013-07-09 | Poland |
| 61d62440-a79c-4178-8245-050f5222e24e | 027 | 2013-07-09 | Poland |
| 2cb41ca4-3e1f-46c4-92bd-8f49b9da0607 | 027 | 2013-07-09 | Poland |
| 4c622de3-7ec6-4756-977d-a523f638c4d0 | 027 | 2013-07-09 | Poland |
| 29a7a92e-7258-4ce2-8c43-1bc6050467f6 | 027 | 2013-07-10 | Poland |
| a0221045-6999-4389-bb8e-a2def142ccf9 | 027 | 2013-07-10 | Poland |
| ece4f8f5-31f6-4ddb-b3f4-c4ed35a221bb | 027 | 2013-07-10 | Poland |
| b892efe6-4f6c-4221-9512-e2bf9876eb91 | 027 | 2013-01-17 | Portugal |
| 796fb9df-40f0-4943-974f-97c8c62816aa | 027 | 2013-07-12 | Portugal |
| c1b49c60-ad12-4e84-8aab-a903dd70e2e1 | 027 | 2013-01-07 | Romania |
| f55dd567-2bcf-42b4-9860-7c64759cea01 | 027 | 2013-01-08 | Romania |
| 855e53c6-305a-4d3d-81f3-5c1b9f06ddd3 | 027 | 2013-01-09 | Romania |
| 3cb69555-bd0e-420c-87bc-bb46d864b94a | 027 | 2013-01-09 | Romania |
| 8496fcee-97ef-48cc-8691-7d98181ee5ce | 027 | 2013-01-09 | Romania |
| e0751a44-96a4-4639-a2e0-2f7814488092 | 027 | 2013-01-09 | Romania |
| dd7f4b51-e5c1-48fb-ba68-1093f6b9d4d9 | 027 | 2013-01-09 | Romania |
| 66e9514f-e716-401b-8bc2-9308f18c7e95 | 027 | 2013-01-10 | Romania |
| f6a583d5-c4d9-411c-9173-18ff6a239319 | 027 | 2013-01-10 | Romania |
| 0d4f2c23-adbc-4647-817f-292298a01355 | 027 | 2013-01-10 | Romania |
| aabf2735-7c8d-4ede-8b20-64de80c8b097 | 027 | 2013-01-10 | Romania |
| 21e03f26-92d2-400d-ad8f-c2c15327a273 | 027 | 2013-01-10 | Romania |
| a912370f-8d26-49c1-b32a-43c3761ca2af | 027 | 2013-01-10 | Romania |
| 56c32b16-f740-4adc-841f-f20dd3c26ed7 | 027 | 2013-01-10 | Romania |
| f1b40349-e975-4d30-950f-bdff1ce9aba3 | 027 | 2013-01-15 | Romania |
| 80a4409f-407c-457a-9726-bccd3396e591 | 027 | 2013-07-01 | Romania |
| 2658201e-61e0-4c6f-9c82-33c512ea8e16 | 027 | 2013-07-02 | Romania |
| d16f671e-c06e-4142-8ea0-00ac4bb47c60 | 027 | 2013-07-04 | Romania |
| a652537f-e703-4356-9537-cf0038494b4e | 027 | 2013-07-04 | Romania |
| ff2f2d73-b6ae-4dcd-aba5-2ef3e9fadfab | 027 | 2013-07-08 | Romania |
| 99b46ed1-38bd-42eb-bcff-fd3446890b9b | 027 | 2013-07-08 | Romania |
| f1af3c44-db7d-468d-9c5a-d18f412d0351 | 027 | 2013-07-09 | Romania |
| 69e91783-112b-4aa9-b619-16a2af942473 | 027 | 2013-07-09 | Romania |
| f109bcbd-48e5-4f89-9b6b-0eeb349aa09c | 027 | 2013-07-09 | Romania |
| c7e83847-e3c9-438b-bd71-03085d4e2a29 | 027 | 2013-07-09 | Romania |
| 98a60a87-125a-4fde-bfa0-92bea738cf59 | 027 | 2013-07-11 | Romania |
| 7c478ec2-4665-4c04-a9ae-e55a678430e5 | 027 | 2013-01-17 | UK |
| c01a402e-bfae-4999-94db-1ce7bc982b3b | 027 | 2013-08-16 | UK |
| 37ae4239-f3b1-4d18-9bc1-4b614beb5a0e | 078 | 2012-12-04 | Austria |
| dc6010a7-dfd1-43ac-8634-07ba3048189d | 078 | 2012-12-05 | France |
| 36ce68ff-d7cf-4217-8d98-107e91c35d3b | 078 | 2013-06-30 | France |
| dcebd0bc-d5b3-43cc-9881-d33ff78ad8aa | 078 | 2013-07-03 | France |
| 93096f72-1dcc-4c6f-96d2-52719cf1a29b | 078 | 2013-01-06 | Germany |
| 8a5a4819-e759-45b2-8c4d-937bc4063d5f | 078 | 2013-01-08 | Germany |
| 4268b7bf-d3f0-47f2-a503-56bfa8fb2a74 | 078 | 2013-01-10 | Germany |
| 2118819b-6b59-45dc-8dfd-b90459c8b695 | 078 | 2013-01-15 | Germany |
| 5d671d6a-f37b-4935-8b7c-630e3578b9af | 078 | 2013-07-03 | Germany |
| a2c08fc5-6cff-4902-8f34-0c07249a4eab | 078 | 2013-07-03 | Germany |
| b6f83637-1fe1-450f-be4e-33401dd983bf | 078 | 2013-07-05 | Germany |
| 233181a9-a2f7-4137-aa9b-38a8c86052dd | 078 | 2013-07-08 | Germany |
| 57a5f566-116d-48b3-9d34-1ac99b3482a6 | 078 | 2013-07-10 | Germany |
| b36da4b4-63db-4e10-a784-5a21607205b9 | 078 | 2013-01-15 | Greece |
| c6755f7e-697c-422a-ab7f-b23689f3d00b | 078 | 2013-01-06 | Ireland |
| 4097a98a-3bbf-42a1-8d1b-0734ef75bcc2 | 078 | 2013-08-05 | Ireland |
| 196696b4-e630-41e9-bb94-c4f43a4c5cb0 | 078 | 2013-01-06 | Italy |
| 3597f2ef-c8d5-4afb-b21e-915216c8d136 | 078 | 2013-01-07 | Italy |
| 439b2836-2bfc-4cb4-b5f2-96a1a9bbf9b4 | 078 | 2013-01-14 | Italy |
| 8ce703d7-e9fc-4ba6-9d07-b1a99675b7ed | 078 | 2013-01-15 | Italy |
| 98e35058-46f3-4363-b7ef-2e16889a1229 | 078 | 2013-07-01 | Italy |
| 2ef7a248-d9c9-4df1-9560-242bf3676f5d | 078 | 2013-07-08 | Italy |
| 7af9d02c-7927-4a56-9335-ba2d348c3f55 | 078 | 2013-07-08 | Italy |
| c10dbbb6-56f4-4afa-9519-b5bd16d64b20 | 078 | 2013-01-08 | Portugal |
| b263a735-6b21-4b70-8ec8-c7721b00ed6f | 078 | 2013-01-17 | Portugal |
| cc8c1189-f7be-4664-b6c8-dbe6818e0579 | 078 | 2013-07-09 | Portugal |
| 7cbad540-efbb-4158-9227-5641bc9194ea | 078 | 2013-07-09 | Spain |
| 834030ec-18cd-46fb-b883-e3ea375c8185 | 078 | 2013-01-10 | UK |
| a604c7cd-ea33-4eb5-83ae-140fed5d6b3e | 078 | 2013-01-15 | UK |
| 1269f0f4-583f-4239-8e70-f7640bd350dc | 078 | 2013-08-05 | UK |
| 66108826-7a8c-4f03-8da1-3b6cd3cde48e | 078 | 2013-08-05 | UK |
| 6385ebe1-a64e-4390-821a-ab1ba3311bf5 | 176 | 2013-01-07 | Czech Republic |
| 5b728dd2-6fae-40cb-9a0d-103c02f335fc | 176 | 2013-01-09 | Czech Republic |
| 735dedf8-5856-4b0b-8148-4b1705b7a4f3 | 176 | 2013-01-14 | Czech Republic |
| 23bed506-bdef-4316-ae8a-969350be4d66 | 176 | 2013-01-14 | Czech Republic |
| 799cf0f8-bc9c-4284-8327-be095c3ae8ac | 176 | 2013-01-14 | Czech Republic |
| 26ace4fe-313c-4398-87c2-a9c5716823e8 | 176 | 2013-01-14 | Czech Republic |
| 0b7a0a5b-9b3b-4ba2-b1a4-5aabc7d334c8 | 176 | 2013-01-15 | Czech Republic |
| 3b35d73c-db0d-4283-a8e8-7dbb0ca7835f | 176 | 2013-01-18 | Czech Republic |
| 72711a33-d4a1-4b8a-913c-cd3e427858b6 | 176 | 2013-07-01 | Czech Republic |
| 7ac75243-28c0-4cf6-8fd9-889354f2b857 | 176 | 2013-07-02 | Czech Republic |
| 2c437dc5-9684-4eb4-a50c-bd1be006e274 | 176 | 2013-07-02 | Czech Republic |
| ce8fe3f1-c679-4722-b775-d586d80ae9a6 | 176 | 2013-07-09 | Czech Republic |
| 75277d56-150b-4bf1-8704-58bce5db8a49 | 176 | 2013-01-14 | Germany |
| ab6ac929-b251-46cd-be4f-53351e9cc754 | 176 | 2013-01-15 | Germany |
| 2397afdb-7286-4d87-8a6a-e6032c0dfab5 | 176 | 2013-07-09 | Germany |
| f80e655b-e858-4a66-9bf4-3ed99ac9d401 | 176 | 2013-07-09 | Germany |
| 1d4509b9-ce9e-479c-9f34-c93e3771f0a5 | 176 | 2013-07-09 | Germany |
| 93a8808e-e0f2-4e67-8cf7-ee44985c72f9 | 176 | 2013-01-10 | Poland |
| 40408d06-ba39-4f3a-b6eb-bdbb967a1c45 | 176 | 2013-07-01 | Poland |
| d8e3d5ba-9a64-46bf-b1b8-82df993b0b39 | 176 | 2013-07-02 | Poland |
| 64be490c-3db3-4862-8da6-a802016fb09c | 176 | 2013-07-02 | Poland |
| 87d95eb1-7794-4bd3-8b0d-45854e4859d8 | 356 | 2013-01-06 | Italy |
| beb19b90-8b8e-4ecf-a979-79d2f0485bdd | 356 | 2013-01-07 | Italy |
| 57ecce95-9ba7-4e94-87b7-dfdf796fdabd | 356 | 2013-01-08 | Italy |
| 7eef40f3-a65e-4837-a278-395227381d99 | 356 | 2013-01-09 | Italy |
| d04494fc-000b-4a79-bff3-919fe8d314a4 | 356 | 2013-01-14 | Italy |
| d3578407-5459-4947-9acb-a53d3fd1dde0 | 356 | 2013-01-14 | Italy |
| 0c166457-d7d2-41aa-ac6f-8753856d3731 | 356 | 2013-01-15 | Italy |
| 1da370a9-9ba4-47e8-9898-95b856f8d425 | 356 | 2013-01-15 | Italy |
| 77b2ea09-2e5b-4c15-8e13-ab04ec13e6c8 | 356 | 2013-07-02 | Italy |
| 1bd860a0-5aaf-4998-afa5-31759584a799 | 356 | 2013-07-02 | Italy |
| 24f4e049-2bb6-40fa-8d45-6bf34521817a | 356 | 2013-07-02 | Italy |
| d7053a2a-824d-4cf5-a2e9-999dfa84684e | 356 | 2013-07-08 | Italy |
| 8d709f2e-24ae-4382-a1f4-32b05b44da6f | 356 | 2013-07-08 | Italy |
| 1a2531c4-8ab4-49cb-a799-8c9369b38673 | 356 | 2013-07-08 | Italy |
| 2017f91e-9f2a-47b6-a247-a91beab10dcd | 356 | 2013-07-09 | Italy |
| 5dd7632f-2643-41ad-986f-e9b770b602f3 | 356 | 2013-07-09 | Italy |
| 81c4d8ed-19b1-4926-947e-4ce95d3a35ce | 356 | 2013-07-09 | Italy |

**Table S3. Sequenced isolates.**
